# Involvement of Ubiquitin-Editing Protein A20 in Modulating Inflammation in Rat Cochlea Associated with Silver Nanoparticle-Induced CD68 Upregulation and TLR4 Activation

**DOI:** 10.1186/s11671-016-1430-9

**Published:** 2016-05-04

**Authors:** Hao Feng, Ilmari Pyykkö, Jing Zou

**Affiliations:** Hearing and Balance Research Unit, Field of Oto-laryngology, School of Medicine, University of Tampere, Medisiinarinkatu 3, 33520 Tampere, Finland; Department of Otolaryngology-Head and Neck Surgery, Center for Otolaryngology-Head and Neck Surgery of Chinese PLA, Changhai Hospital, Second Military Medical University, Shanghai, China

## Abstract

Silver nanoparticles (AgNPs) were shown to temporarily impair the biological barriers in the skin of the external ear canal, mucosa of the middle ear, and inner ear, causing partially reversible hearing loss after delivery into the middle ear. The current study aimed to elucidate the molecular mechanism, emphasizing the TLR signaling pathways in association with the potential recruitment of macrophages in the cochlea and the modulation of inflammation by ubiquitin-editing protein A20. Molecules potentially involved in these signaling pathways were thoroughly analysed using immunohistochemistry in the rat cochlea exposed to AgNPs at various concentrations through intratympanic injection. The results showed that 0.4 % AgNPs but not 0.02 % AgNPs upregulated the expressions of CD68, TLR4, MCP1, A20, and RNF11 in the strial basal cells, spiral ligament fibrocytes, and non-sensory supporting cells of Corti’s organ. 0.4 % AgNPs had no effect on CD44, TLR2, MCP2, Rac1, myosin light chain, VCAM1, Erk1/2, JNK, p38, IL-1β, TNF-α, TNFR1, TNFR2, IL-10, or TGF-β. This study suggested that AgNPs might confer macrophage-like functions on the strial basal cells and spiral ligament fibrocytes and enhance the immune activities of non-sensory supporting cells of Corti’s organ through the upregulation of CD68, which might be involved in TLR4 activation. A20 and RNF11 played roles in maintaining cochlear homeostasis via negative regulation of the expressions of inflammatory cytokines.

**Electronic supplementary material:**

The online version of this article (doi:10.1186/s11671-016-1430-9) contains supplementary material, which is available to authorized users.

## Background

With the rapid development of nanotechnology and increasing applications of engineered nanomaterials in our daily lives, their potential safety issues have become a serious concern in public health. The rat ear model has been applied to investigate the impact of silver nanoparticles (AgNPs) on the permeability of biological barriers in the skin, mucosa, and inner ear that is analogous to the nervous system (e.g. the brain and spinal cord) [1]. Previous research showed that AgNPs led to hyaluronan accumulation in the cochlea, impaired biological barriers in the skin of the external ear canal, mucosa of the middle ear, and inner ear, and consequently caused hearing loss after delivery into the middle ear [1–3]. Hyaluronan acts as an endogenous pathogen-associated molecular pattern (PAMP) in response to hazardous signals through binding hyaluronan-binding proteins (hyaladherins) including toll-like receptors 2/4 (TLR2/4), CD44, receptor for hyaluronan-mediated motility, and tumour necrosis factor-α (TNF-α)-stimulated glycoprotein-6 [4–7]. Among them, TLR2/4 is a category of mammalian homologues of *Drosophila* Toll proteins that are of great importance for innate host defence. They belong to the pattern recognition receptors (PRRs) that specifically recognize and respond to an expansive variety of PAMPs [8]. Moreover, TLR4 is responsible for sensing danger/damage-associated molecular patterns (DAMPs) and conferring immunostimulatory activity [9]. The activation of TLRs initiates the upregulation of transcription factors (e.g. nuclear factor-κB (NF-κB) and activator protein-1) that play pivotal roles in producing inflammatory molecules (e.g. interleukin-1β (IL-1β), interleukin-6 (IL-6), and TNF-α together with its receptors TNFRs), chemokines (e.g. monocyte chemoattractant proteins (MCPs)), and reactive oxygen/nitrogen species, leading to inflammatory diseases [10–12].

Several proteins that are implicated in mediating TLR signaling attenuation have been identified such as the ubiquitin-editing protein A20 [13–15]. A20 acts as a negative effector in regulating TLR-mediated inflammatory response, and its overexpression inhibits TLR2- and TLR4-mediated IL-8 syntheses in airway epithelial cells [16]. A20 loss elevates the levels of NF-κB-regulated inflammatory cytokines and causes spontaneous cerebral inflammation [17]. RING finger protein 11 (RNF11), a critical component of A20, is indicated as one of the key negative regulators in controlling the NF-κB signaling pathway. RNF11 was shown to protect microglia irritated by lipopolysaccharide through manipulating the NF-κB signaling pathway [18]. RNF11 knockdown in the monocytes led to persistent TNF- and lipopolysaccharide-mediated NF-κB signaling activation and upregulated NF-κB-associated inflammatory gene transcripts [18, 19].

As another important hyaladherin, CD44 is capable of recruiting monocytes from the peripheral blood upon hyaluronan binding [20]. Further study has revealed that weakened interaction between CD44 and hyaluronan decreases the production of MCPs and consequently undermines the recruitment of mononuclear cells [21]. MCPs are a family of small heparin-binding, positively charged chemokines that play an indispensable role in controlling cell behaviour in response to exogenous stimulation. They are crucial in triggering the mobilization and migration of immunocompetent cells such as monocytes, neutrophils, lymphocytes, and dendritic cells along the bone marrow sinusoids that frequently anastomose with capillaries and in directing them into the inflamed tissues [22]. In the inner ear, spiral ligament fibrocytes act as the primary immune sensors in response to lipopolysaccharide, involving TLR2-dependent NF-κB signaling activation and MCP1 upregulation and resulting in monocyte migration and consequential infiltration [23, 24].

Adhesion molecules play a critical role in mediating leukocyte immobilization as a result of anchoring [25]. Among them, vascular cell adhesion molecule 1 (VCAM1) enables rolling monocytes along the microvascular wall at a far slower velocity to adhere to the endothelial cells [26]. Rac1, a member of Rho-like small GTPase, mediated by the phosphorylation of myosin light chain protein, facilitates actin cytoskeletal remodelling and modulates tight junctional proteins (e.g. occludin and claudin). The breakdown of tight junction in the microvascular wall enables the leukocytes to infiltrate into the targeting site [27–29]. The extracellular signal-regulated kinases 1/2 (Erk1/2), c-Jun N-terminal kinases 1/2/3 (JNK1/2/3) (also known as stress-activated protein kinases), and p38 isoforms (α, β, γ, and δ) that belong to the MAPKs family are considered to be the elementary components of cellular signaling transduction underlying leukocyte locomotion and endothelial cell activities [30, 31].

Migrated monocytes can differentiate into macrophages. Plasticity and flexibility are the key features of macrophages and reflect their activation states [32]. Activated macrophages have distinctive functional phenotypes that are similar to the Th1/Th2 polarization paradigm of T lymphocytes and can be defined as M1 and M2. M1 induced by Th1 signature cytokines (e.g. interferon-γ (IFN-γ) and TNF-α), which are associated with the TLR-dependent signaling pathway, has the ability of upregulating genes involved in cell-biased immunity, enhancing antigen presentation, and producing a distinctive array of inflammatory cytokines (e.g. IL-1β, IL-6, and TNF-α). M2 induced by Th2 signature cytokines (e.g. IL-4 and IL-13) plays an important role in immune suppression, anti-inflammation (e.g. interleukin-10 (IL-10)), tissue regeneration, and wound healing (e.g. transforming growth factor-β (TGF-β) and vascular endothelial growth factor (VEGF)) [33, 34].

The current study aimed to elucidate the exact mechanism of AgNP-induced biological barrier functional changes in the inner ear. We exposed the rat inner ear to AgNPs and hypothesized that TLR signaling pathways were involved in AgNP-induced hearing loss in association with the potential recruitment of macrophages in the rat cochlea. A20 might play a role in regulating the downstream signaling of TLR pathways. Molecules potentially involved in these signaling pathways were thoroughly analysed using immunohistochemistry in the rat cochlea after AgNP exposure.

## Methods

### Animal and AgNPs

Ten albino male Sprague-Dawley rats weighing between 250 and 300 g were kept at an ambient temperature of 20–22 °C with a relative humidity of 50 ± 5 % under a 12/12-h light/dark cycle in the experimental animal unit, University of Tampere. The experiments were performed under general anaesthesia with a mixture of 0.5 mg/kg medetomidine hydrochloride (Domitor®, Orion, Espoo, Finland) and 75 mg/kg ketamine hydrochloride (Ketalar®, Pfizer, Helsinki, Finland) administered via intraperitoneal injection, followed by intramuscular injection of enrofloxacin (Baytril®vet, Orion, Turku, Finland) at a dose of 10 mg/kg to prevent potential infection. The animals’ eyes were protected by carbomer (Viscotears®, Novartis Healthcare A/S, Denmark). All procedures in the study complied with the local ethics committee standards (permission number: ESAVI/3033/04.10.03/2011) and were conducted in accordance with European Legislation. The AgNPs (Colorobbia, Firenze, Italy) used in this study were highly faceted with a mean size of 21 ± 8 nm using transmission electron microscope. The mean hydrodynamic size of the particles was 117 ± 24 nm when suspended in deionized water (dH_2_O) using dynamic light scattering, and the zeta potential was measured to be −20 ± 9 mV [2]. More results for the characterization could be referred to our previous study [2].

### AgNP Administration

After anaesthetization, 40 μl of either 0.4 (*n* = 5) or 0.02 % (*n* = 5) AgNPs were injected into the middle ear cavity under an operating microscope (OPMI1-F, Carl Zeiss, Jena, Germany) according to a previously reported procedure [1–3]. The tested concentrations were selected according to the auditory brainstem response results showing that 0.4 % AgNPs caused reversible hearing loss that partially recovered at the seventh day, while 0.02 % AgNPs only induced hearing loss at 32 kHz that returned to the baseline at the seventh day. Moreover, micro-CT scanning displayed that 0.4 % AgNPs caused an obvious middle ear infiltration that was absent in the rats exposed to 0.02 % AgNPs [1–3]. The contralateral ear (*n* = 10) received dH_2_O under the same circumstances and was used as a negative control.

### Sample Preparation

On the seventh day post-injection, the anaesthetized rats were perfused with 0.01 M pH 7.4 phosphate-buffered saline (PBS) containing 0.6 % (*v*/*v*) heparin (LEO Pharma A/S, Ballerup, Denmark) via a cardiac approach followed by 4 % paraformaldehyde (Merck, Espoo, Finland) to fix the head. The bullae were isolated after decapitation and decalcified using 10 % EDTA (Sigma-Aldrich, Steinheim, Germany) in the following 4 weeks with weekly solution changes. A standard procedure for paraffin embedding and tissue block was conducted in accordance with the protocol in a previous study [3].

### Immunofluorescence Staining

The procedure for immunofluorescence staining was in accordance with the protocol in a previous study [3]. The primary antibodies used in the assay were hosted in rabbit and were anti-CD68 (1:200, Abcam, UK), anti-CD44 (1:400, Abcam, UK), anti-TLR2 (1:250, Novus Biologicals, UK), anti-TLR4 (1:200, Novus Biologicals, UK), anti-MCP1 (1:4000, Novus Biologicals, UK), anti-MCP2 (1:200, GeneTex, USA), anti-Rac1 (1:800, Abcam, UK), anti-myosin light chain (1:100, Cell Signaling Technology, USA), anti-VCAM1 (1:50, Proteintech, USA), anti-Erk1/2 (1:400, Abcam, UK), anti-JNK (1:100, Cell Signaling Technology, USA), anti-p38 (1:100, Cell Signaling Technology, USA), anti-TNF-α (1:800, Abcam, UK), anti-TNFR1 (1:500, Abcam, UK), anti-TNFR2 (1:50, Abcam, UK), anti-IL-1β (1:400, Novus Biologicals, UK), anti-IL-10 (1:400, Abbiotec, USA), anti-TGF-β (1:500, Abcam, UK), anti-A20 (1:200, Sigma-Aldrich, USA), and anti-RNF11 (1:100, Abcam, UK). Briefly, the slices were incubated with the primary antibodies listed above at 4 °C overnight followed by Alexa Fluor® 488 Goat Anti-Rabbit IgG (1:200, diluted with 0.1 % BSA, Life Technologies™, New York, USA) as a secondary antibody at room temperature for 1 h in a dark environment. The nuclei were counterstained with 10 μg/ml DAPI (Life Technologies™, New York, USA) at room temperature for 10 min, and the slides were mounted for confocal microscopy with anti-quenching fluoromount (Sigma-Aldrich, St. Louis, USA). In the negative control slices, the primary antibodies were replaced with 0.1 % BSA (dissolved in 0.01 M PBS pH 7.4; Sigma-Aldrich, St. Louis, USA).

### Immunostaining Visualized by 3,3′-Diaminobenzidine

After deparaffinization and hydration, the slices were immersed in 3 % H_2_O_2_-methanol at room temperature for 30 min. After rinsing with PBS for 2 × 2 min, the slices were digested with 0.1 % trypsin (dissolved in 0.01 M PBS pH 7.4; Sigma-Aldrich, St. Louis, USA) at 37 °C for 30 min. After rinsing with 0.1 % PBS-Tween® 20 (diluted in 0.01 M PBS pH 7.4; Sigma-Aldrich, St. Louis, USA) for 3 × 2 min, the slices were incubated with 10 % normal goat serum (Invitrogen, Paisley, UK) at room temperature for 30 min followed by the primary antibodies listed above at 4 °C overnight. After rinsing with 0.1 % PBS-Tween® 20 for 3 × 2 min, the slices were incubated with biotinylated goat anti-rabbit IgG at a dilution of 1:100 (Vector Laboratories Ltd., Peterborough, UK) at room temperature for 1 h. After rinsing with 0.1 % PBS-Tween® 20 for 3 × 2 min, the slices were incubated with the streptavidin-biotin-peroxidase complex (Vector Laboratories Ltd., Peterborough, UK) at 37 °C for 1 h. After rinsing with 0.1 % PBS-Tween® 20 for 3 × 5 min, antibody binding was visualized by 3,3′-diaminobenzidine using the DAB Peroxidase Substrate Kit (Vector Laboratories Ltd., Peterborough, UK) at room temperature for 5 min. Alternatively, the nuclei were counterstained using Harris’s solution (Merck, Darmstadt, Germany). Dehydration and vitrification were completed by a standard protocol (70 % alcohol 10 s, 94 % alcohol 2 × 10 s, absolute alcohol 2 × 1 min, and xylene 3 × 3 min). The slides were mounted for light microscopy with Clarion™ Mounting Medium (Sigma-Aldrich, St. Louis, USA). Slices for negative controls were prepared after the replacement of primary antibodies with 0.1 % BSA (dissolved in 0.01 M PBS pH 7.4; Sigma-Aldrich, St. Louis, USA). The staining intensities (shown by the greyscale value that was inversely correlated with the staining intensity) in the strial basal cells, spiral ligament fibrocytes, and spiral ganglion cells were measured and semi-quantified using ImageJ 1.45S software (NIH, Bethesda, USA).

### Confocal and Light Microscopies

The samples from immunofluorescence staining were observed and images obtained under a Nikon microscope (ECLIPSE Ti) combined with an Andor confocal system installed with Andor iQ 2.8 software (Andor Technology, Belfast, UK). The excitation lasers were 405 (blue excitation) and 488 nm (green excitation) from an Andor Laser Combiner system, and the corresponding emission filters were 450–465 (DAPI) and 525/50 nm (FITC), respectively. The immunostained samples visualized by 3,3′-diaminobenzidine were observed under a light microscope (LEICA DM 2000, Espoo, Finland), and images were digitally photographed using a camera video (Olympus DP 25, Tokyo, Japan) with the cellSens Dimension 1.6 Olympus software (Olympus Corporation, Tokyo, Japan) installed.

### Analysis and Statistics

Statistical analyses were performed using the IBM® SPSS® Statistics Version 20 software package (SPSS Inc., Chicago, USA). One-way ANOVA was used to compare the staining intensities for CD68, TLR2, TLR4, MCP1, MCP2, A20, and RNF11 in the designated structures of different cochlear turns among the cochleae exposed to 0.4 % AgNPs, 0.02 % AgNPs, and dH_2_O. The LSD post hoc test was used to evaluate the pairwise difference. The independent sample *t* test was used to compare the staining intensities for CD44, Rac1, Erk1/2, IL-1β, IL-10, and TGF-β in the designated structures of different cochlear turns between the cochleae exposed to 0.4 % AgNPs and dH_2_O. A value of *p* < 0.05 indicated that the difference was statistically significant.

## Results

### AgNPs Augment the Sensitivity and Chemotactic Proteins of Cochlear Cells

**Fig. 1:**
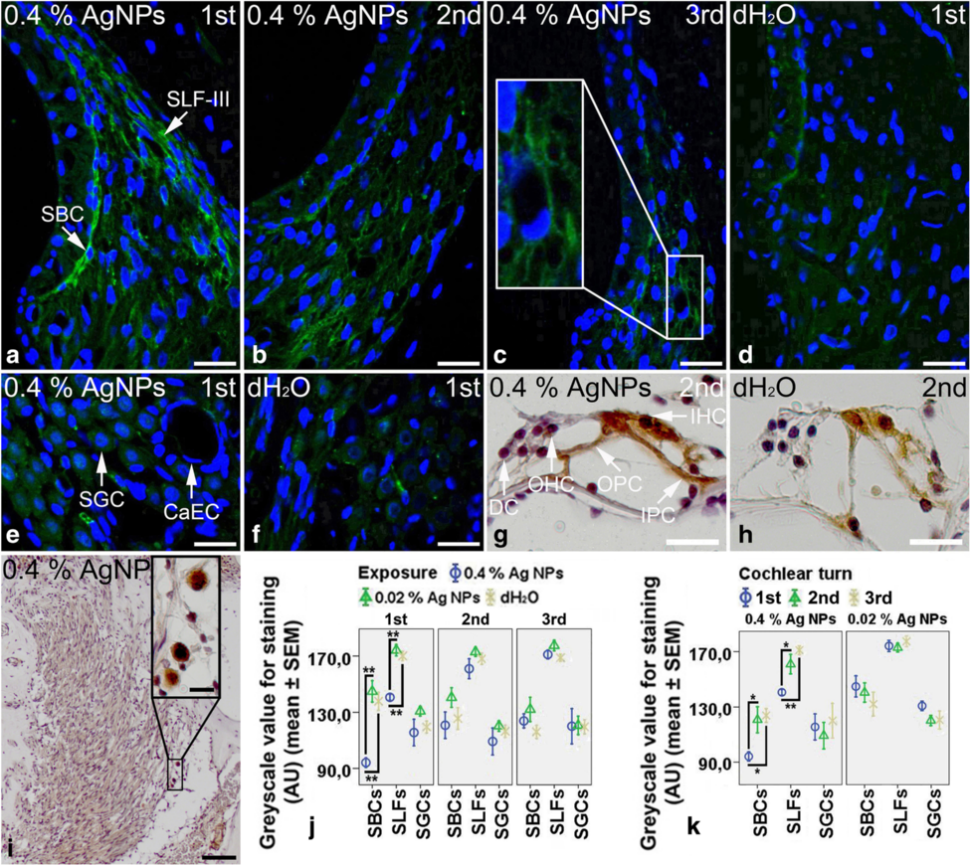
CD68^+^ cells in the rat cochlea 7 days post-intratympanic injection of 0.4 % AgNPs shown by immunofluorescence confocal microscopy or immunohistochemistry. In the cochleae exposed to dH_2_O, the inner hair cells (IHCs) and pillar cells (PCs) of Corti’s organ (CO) showed moderate staining, while the outer hair cells (OHCs) and Deiters’ cells (DCs) demonstrated extremely weak staining (**h**). The strial basal cells (SBCs), spiral ligament fibrocytes (SLFs), and spiral ganglion cells (SGCs) exhibited mild staining (**d**, **f**). In the cochleae exposed to 0.4 % AgNPs, the SBCs and SLFs (mainly type III) in the first turn (**a**) and the IHCs and PCs of CO (**g**) displayed more intensive staining. Sparse ramified cells (**c**) and mononuclear cells (**i**) with CD68 staining were identified in the spiral ligament and the modiolus, respectively. However, the SBCs and SLFs in the second and third turns (**b**, **c**), SGCs (**e**), capillary endothelial cells (CaECs) (**e**), OHCs, and DCs (**g**) did not show any changes. Comparisons of staining intensity are shown in **j** and **k**. Scale bar = 30 μm in **a**–**f**, 20 μm in **g**, **h**, and the magnified image in **i**, and 80 μm in **i**

In the cochleae exposed to dH_2_O, the inner hair cells and pillar cells of Corti’s organ showed moderate staining for CD68, while the outer hair cells and Deiters’ cells demonstrated extremely weak staining for CD68 (Fig. 1h). The strial basal cells, spiral ligament fibrocytes, and spiral ganglion cells exhibited mild staining for CD68 (Fig. 1d, f). In the cochlear lateral wall, 0.4 % AgNPs intensified CD68 staining remarkably in the strial basal cells (*p* < 0.01, post hoc test) and spiral ligament fibrocytes (mainly type III) (*p* < 0.01, post hoc test) in the first turn (Fig. 1a). However, no enhanced staining was observed in cells in the second and third turns (Fig. 1b, c) (*p* > 0.05, post hoc test). In the CD68^+^ cell population, sparse ramified cells and mononuclear cells were identified in the spiral ligament and the modiolus, respectively (Fig. 1c, i). In Corti’s organ, 0.4 % AgNPs increased CD68 staining in the inner hair cells and pillar cells but not in the outer hair cells and Deiters’ cells (Fig. 1g). In the spiral ganglion cells and capillary endothelial cells, 0.4 % AgNPs did not alter CD68 staining in all turns (Fig. 1e) (*p* > 0.05, post hoc test). The 0.02 % AgNPs had no influence on CD68 staining in the aforementioned cells in all turns (images not shown) (*p* > 0.05, post hoc test).

In the cochleae exposed to dH_2_O, the strial intermediate cells, strial basal cells, spiral ligament fibrocytes, spiral ganglion cells, and outer hair cells, pillar cells, and Deiters’ cells of Corti’s organ showed intensive staining for CD44 (Additional file 1: Figure S1B, S1D, and S1F), while the inner hair cells demonstrated mild staining for CD44 (Additional file 1: Figure S1F). 0.4 % AgNPs had no influence on the staining in the aforementioned cells in all turns (Additional file 1: Figure S1A, S1C, and S1E) (*p* > 0.05, independent sample *t* test).

**Fig. 2:**
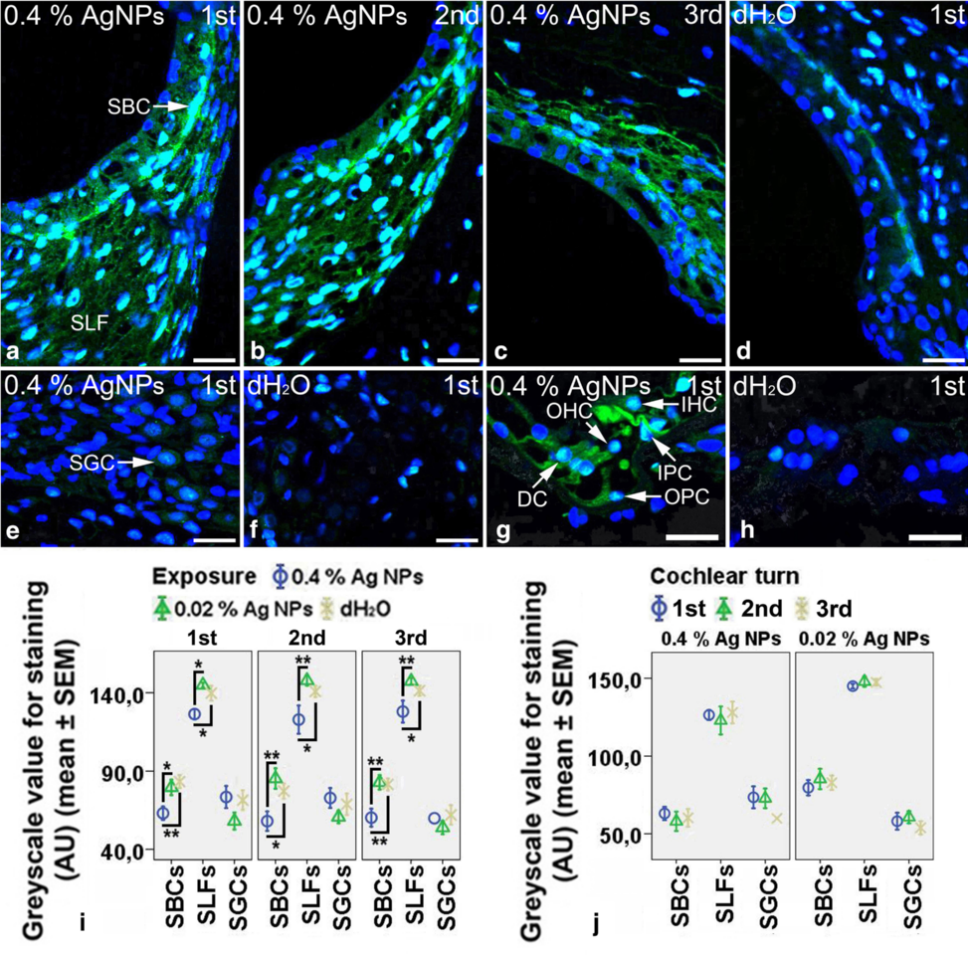
TLR4^+^ cells in the rat cochlea 7 days post-intratympanic injection of 0.4 % AgNPs shown by immunofluorescence confocal microscopy or immunohistochemistry. In the cochleae exposed to dH_2_O, the strial basal cells (SBCs) and spiral ligament fibrocytes (SLFs) showed mild staining (**d**), while the spiral ganglion cells (SGCs), hair cells (HCs), pillar cells (PCs), and Deiters’ cells (DCs) of Corti’s organ (CO) demonstrated extremely weak staining (**f**, **h**). In the cochleae exposed to 0.4 % AgNPs, the SBCs and SLFs exhibited more intensive staining that was independent of the cochlear turn (**a**–**c**). In CO, the inner hair cells (IHCs), PCs, and DCs displayed more intensive staining, but the outer hair cells (OHCs) did not (**g**). However, the SGCs did not show any changes (**e**). Comparisons of staining intensity are shown in **i** and **j**. Scale bar = 30 μm

In the cochleae exposed to dH_2_O, the strial basal cells, spiral ligament fibrocytes (mainly type II), spiral ganglion cells, and inner hair cells and pillar cells of Corti’s organ showed intensive staining for TLR2 (Additional file 2: Figure S2B, S2D, and S2F), while the outer hair cells and Deiters’ cells displayed extremely weak staining for TLR2 (Additional file 2: Figure S2F). The strial basal cells and spiral ligament fibrocytes demonstrated mild staining for TLR4 (Fig. 2d), while the spiral ganglion cells and hair cells, pillar cells, and Deiters’ cells of Corti’s organ exhibited extremely weak staining for TLR4 (Fig. 2f, h). In the cochleae exposed to 0.4 % AgNPs, the outer hair cells and Deiters’ cells of Corti’s organ showed more intensive staining for TLR2 (Additional file 2: Figure S2E). However, the strial basal cells, spiral ligament fibrocytes, and spiral ganglion cells did not show any changes in the staining of TLR2 in all turns (Additional file 2: Figure S2A and S2C) (*p* > 0.05, one-way ANOVA) nor in the inner hair cells and pillar cells (Additional file 2: Figure S2E). The strial basal cells (*p* < 0.05 in the first and second turns and *p* < 0.01 in the third turn, one-way ANOVA) and spiral ligament fibrocytes (Fig. 2a–c) (*p* < 0.05 in the first, second, and third turns, one-way ANOVA) demonstrated more intensive staining for TLR4 that was independent of the cochlear turn (*p* > 0.05, one-way ANOVA). The inner hair cells, pillar cells, and Deiters’ cells displayed more intensive staining for TLR4, but the outer hair cells did not (Fig. 2g). However, the spiral ganglion cells did not show any changes (Fig. 2e). The 0.02 % AgNPs had no influence on the staining of TLR2 and TLR4 in the aforementioned cells in all turns (images not shown) (*p* > 0.05, one-way ANOVA).

**Fig. 3:**
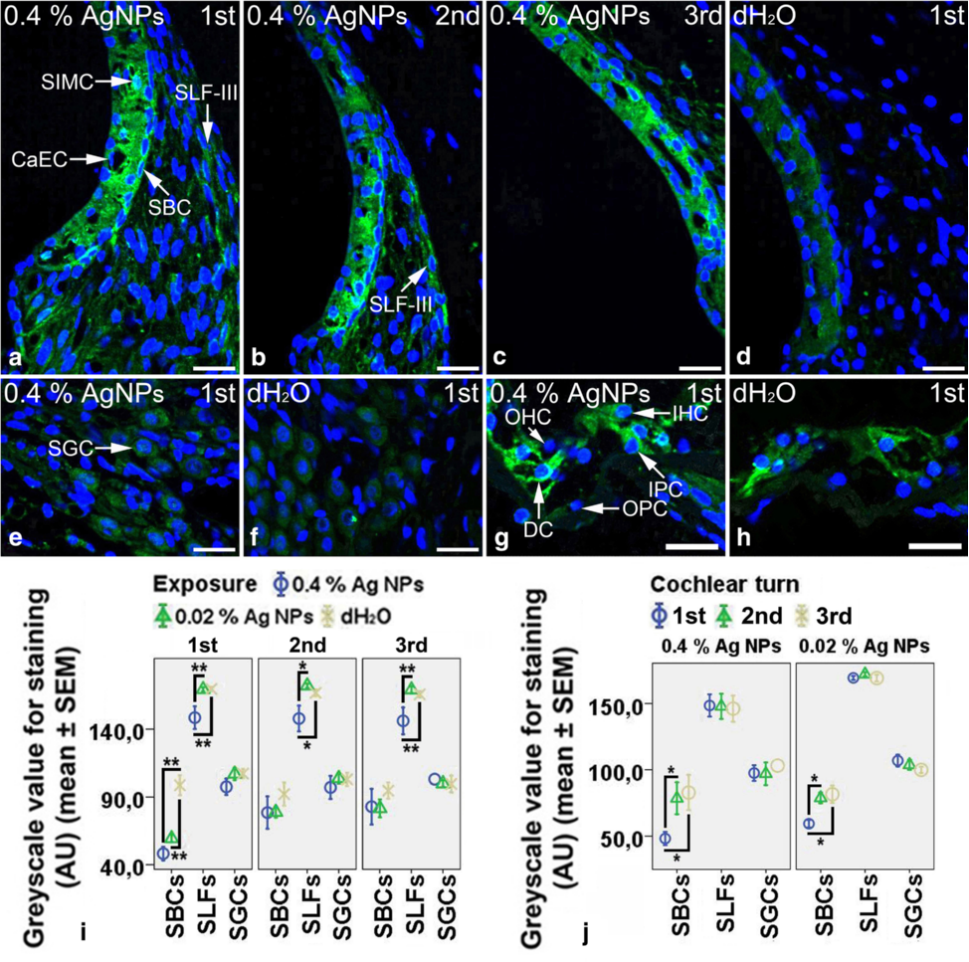
MCP1^+^ cells in the rat cochlea 7 days post-intratympanic injection of 0.4 % AgNPs shown by immunofluorescence confocal microscopy or immunohistochemistry. In the cochleae exposed to dH_2_O, the Deiters’ cells (DCs) of Corti’s organ (CO) showed intensive staining, while the inner hair cells (IHCs) and inner pillar cells (IPCs) exhibited moderate staining (**h**). The strial intermediate cells (SIMCs), strial basal cells (SBCs), spiral ganglion cells (SGCs), and outer hair cells (OHCs) and outer pillar cells (OPCs) of CO demonstrated mild staining, while the spiral ligament fibrocytes (SLFs) displayed extremely weak staining (**d**, **f**, **h**). In the cochleae exposed to 0.4 % AgNPs, the SLFs showed more intensive staining that was independent of the cochlear turn, while the SIMCs, SBCs, and capillary endothelial cells (CaECs) demonstrated more intensive staining in the first turn (**a**–**c**). In CO, the IPCs and DCs exhibited more intensive staining, but the hair cells (HCs) and OPCs did not (**g**). However, the SGCs did not show any changes (**e**). Comparisons of staining intensity are shown in **i** and **j**. Scale bar = 30 μm

In the cochleae exposed to dH_2_O, the Deiters’ cells of Corti’s organ showed intensive staining for MCP1, while the inner hair cells and inner pillar cells exhibited moderate staining for MCP1 (Fig. 3h). The strial intermediate cells, strial basal cells, spiral ganglion cells, outer hair cells, and outer pillar cells demonstrated mild staining for MCP1 (Fig. 3d, f, h), while the spiral ligament fibrocytes displayed extremely weak staining for MCP1 (Fig. 3d). Unexpectedly, the strial basal cells, spiral ligament fibrocytes, spiral ganglion cells, and the hair cells, pillar cells, and Deiters’ cells of Corti’s organ showed intensive staining for MCP2 (Additional file 3: Figure S3B, S3D, and S3F). In the cochleae exposed to 0.4 % (Fig. 3a) and 0.02 % AgNPs (image not shown), the strial intermediate cells, capillary endothelial cells, and strial basal cells (*p* < 0.01, one-way ANOVA) in the first turn demonstrated more intensive staining for MCP1. However, the spiral ligament fibrocytes (mainly type III) in the cochleae exposed to 0.4 % AgNPs (Fig. 3a–c) (*p* < 0.01 in the first and third turns and *p* < 0.05 in the second turn, one-way ANOVA) showed more intensive staining for MCP1 that was independent of the cochlear turn (*p* > 0.05, one-way ANOVA). In addition, 0.4 % AgNPs increased MCP1 staining in the inner pillar cells and Deiters’ cells of Corti’s organ (Fig. 3g). However, the spiral ganglion cells did not show any changes (Fig. 3e) (*p* > 0.05, one-way ANOVA). Neither 0.4 % nor 0.02 % AgNPs affected the staining of MCP2 in the aforementioned cells in all turns (images not shown) (*p* > 0.05, one-way ANOVA).

### AgNPs had no Effect on the Expressions of Tight Junction-Associated Proteins Including Rac1, Myosin Light Chain, VCAM1, and MAPK Signaling Proteins

In the cochleae exposed to dH_2_O, the strial intermediate cells, strial basal cells, spiral ganglion cells, and hair cells, pillar cells, and Deiters’ cells of Corti’s organ showed intensive staining for Rac1 (Additional file 4: Figure S4B, S4D, and S4F), while the spiral ligament fibrocytes (mainly type II) demonstrated moderate staining for Rac1 (Additional file 4: Figure S4B). The spiral ganglion cells and inner pillar cells of Corti’s organ exhibited moderate staining for myosin light chain (Additional file 5: Figure S5D and S5F), while the hair cells, outer pillar cells, and Deiters’ cells displayed mild staining for myosin light chain (Additional file 5: Figure S5F). The strial basal cells and spiral ligament fibrocytes showed extremely weak staining for myosin light chain (Additional file 5: Figure S5B). The strial basal cells, spiral ligament fibrocytes, spiral ganglion cells, and hair cells, pillar cells, and Deiters’ cells of Corti’s organ showed extremely weak staining for VCAM1 (Additional file 6: Figure S6B, S6D, and S6F), JNK (Additional file 7: Figure S8B, S8F, and S8J), and p38 (Additional file 7: Figure S8D, S8H, and S8L). However, the strial intermediate cells, strial basal cells, spiral ligament fibrocytes, spiral ganglion cells, and hair cells, pillar cells, and Deiters’ cells of Corti’s organ showed intensive staining for Erk1/2 (Additional file 8: Figure S7B, S7D, and S7F). 0.4 % AgNPs had no influence on the staining of Rac1 (Additional file 4: Figure S4A, S4C, and S4E) (*p* > 0.05, independent sample *t* test), myosin light chain (Additional file 5: Figure S5A, S5C, and S5E), VCAM1 (Additional file 6: Figure S6A, S6C, and S6E), Erk1/2 (Additional file 8: Figure S7A, S7C, and S7E) (*p* > 0.05, independent sample *t* test), JNK (Additional file 7: Figure S8A, S8E, and S8I), and p38 (Additional file 7: Figure S8C, S8G, and S8K) in the aforementioned cells in all turns.

### AgNPs Upregulated the Expressions of Ubiquitin-Editing Proteins A20 and RNF11 Without Affecting the Expressions of Inflammatory Cytokines

In the cochleae exposed to dH_2_O, the spiral ganglion cells, inner hair cells, and inner pillar cells of Corti’s organ showed mild staining for TNF-α (Additional file 9: Figure S9H and S9N), while the strial basal cells, spiral ligament fibrocytes, outer pillar cells, outer hair cells, and Deiters’ cells demonstrated extremely weak staining for TNF-α (Additional file 9: Figure S9B and S9N). The strial intermediate cells, strial basal cells, and spiral ganglion cells exhibited mild staining for TNFR1 (Additional file 9: Figure S9D and S9J), while the spiral ligament fibrocytes, hair cells, pillar cells, and Deiters’ cells displayed extremely weak staining for TNFR1 (Additional file 9: Figure S9D and S9P). The strial intermediate cells and strial basal cells showed mild staining for TNFR2 (Additional file 9: Figure S9F), while the spiral ligament fibrocytes, spiral ganglion cells, hair cells, pillar cells, and Deiters’ cells demonstrated extremely weak staining for TNFR2 (Additional file 9: Figure S9F, S9L, and S9R). The strial basal cells, spiral ganglion cells, and pillar cells of Corti’s organ exhibited intensive staining for IL-1β, while the spiral ligament fibrocytes (mainly type II) and inner hair cells displayed mild staining for IL-1β (Additional file 10: Figure S10B, S10D, and S10F). The outer hair cells and Deiters’ cells showed extremely weak staining for IL-1β (Additional file 10: Figure S10F). 0.4 % AgNPs had no influence on the staining of TNF-α (Additional file 9: Figure S9A, S9G, and S9M), TNFR1 (Additional file 9: Figure S9C, S9I, and S9O), TNFR2 (Additional file 9: Figure S9E, S9K, and S9Q), and IL-1β (Additional file 10: Figure S10A, S10C, and S10E) (*p* > 0.05, independent sample *t* test) in the aforementioned cells in all turns.

In the cochleae exposed to dH_2_O, the spiral ganglion cells showed intensive staining for IL-10 (Additional file 11: Figure S11F), while the pillar cells of Corti’s organ demonstrated mild staining for IL-10 (Additional file 11: Figure S11J). The strial basal cells, spiral ligament fibrocytes, hair cells, and Deiters’ cells exhibited extremely weak staining for IL-10 (Additional file 11: Figure S11B and S11J). The spiral ganglion cells and pillar cells of Corti’s organ displayed intensive staining for TGF-β (Additional file 11: Figure S11H and S11L), while the strial basal cells, spiral ligament fibrocytes, and inner hair cells demonstrated mild staining for TGF-β (Additional file 11: Figure S11D and S11L). The outer hair cells and Deiters’ cells showed extremely weak staining for TGF-β (Additional file 11: Figure S11L). 0.4 % AgNPs had no influence on the staining of IL-10 (Additional file 11: Figure S11A, S11E, and S11I) (*p* > 0.05, independent sample *t* test) and TGF-β (Additional file 11: Figure S11C, S11G, and S11K) (*p* > 0.05, independent sample *t* test) in the aforementioned cells in all turns.

**Fig. 4:**
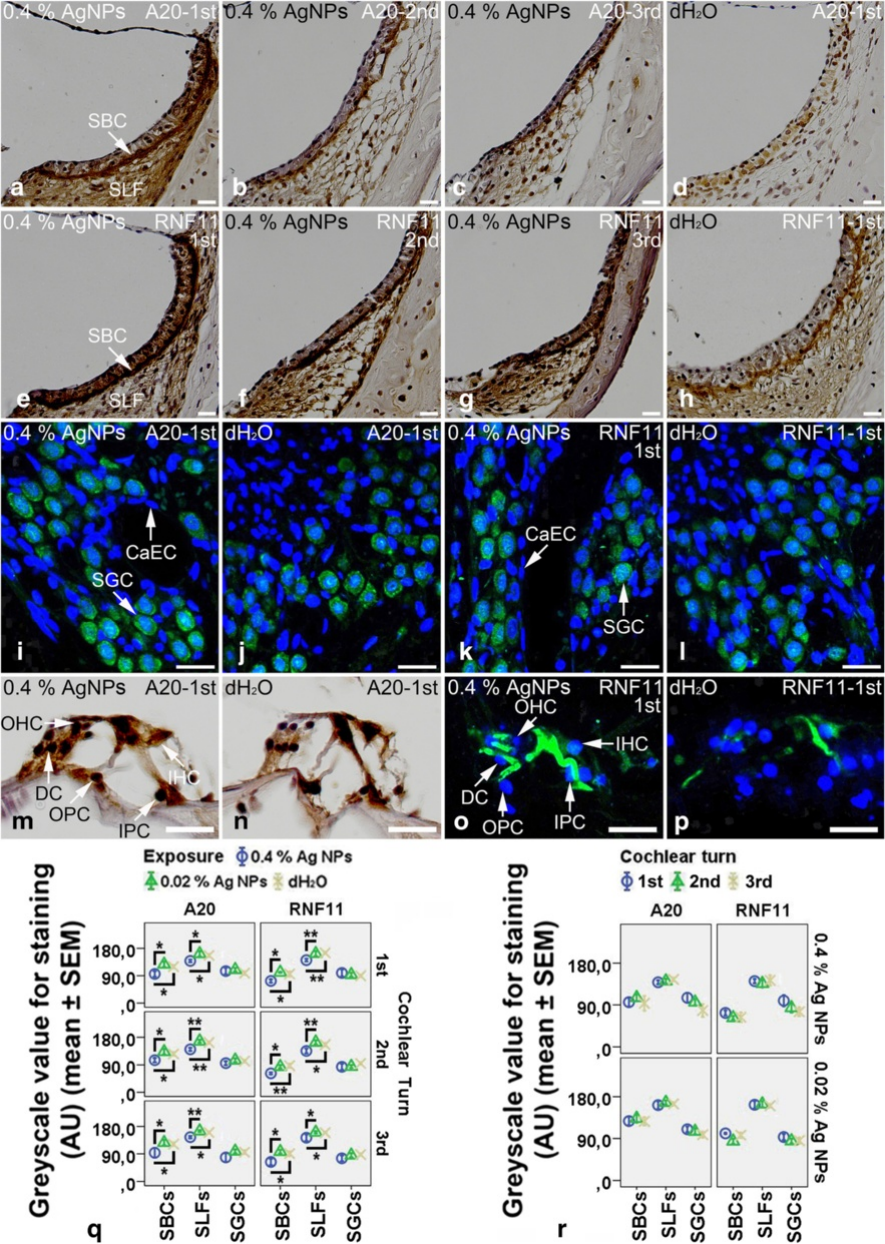
A20^+^ and RNF11^+^ cells in the rat cochlea 7 days post-intratympanic injection of 0.4 % AgNPs shown by immunofluorescence confocal microscopy or immunohistochemistry. In the cochleae exposed to dH_2_O, the spiral ganglion cells (SGCs), inner hair cells (IHCs), pillar cells (PCs), and Deiters’ cells (DCs) of Corti’s organ (CO) showed intensive staining for A20 (**j**, **n**), while the strial basal cells (SBCs), spiral ligament fibrocytes (SLFs), and outer hair cells (OHCs) demonstrated mild staining for A20 (**d**, **n**). The SBCs, SGCs, and inner pillar cells (IPCs) of CO exhibited intensive staining for RNF11, while the SLFs, hair cells (HCs), and outer pillar cells (OPCs) displayed mild staining for RNF11 (**h**, **l**, **p**). The DCs showed extremely weak staining for RNF11 (**p**). In the cochleae exposed to 0.4 % AgNPs, the SBCs and SLFs demonstrated more intensive staining for A20 and RNF11 that was independent of the cochlear turn (**a**–**c**, **e**–**g**). In CO, the OHCs and DCs displayed more intensive staining for A20 (**m**), the OPCs and DCs exhibited more intensive staining for RNF11 (**o**). However, the SGCs and capillary endothelial cells (CaECs) did not show any changes in the staining of A20 and RNF11 (**i**, **k**). Comparisons of staining intensity are shown in **q** and **r**. Scale bar = 50 μm in **a**–**h**, 20 μm in **m**, **n**, and 30 μm in **i**–**l**, **o**, **p**

In the cochleae exposed to dH_2_O, the spiral ganglion cells, inner hair cells, pillar cells, and Deiters’ cells of Corti’s organ showed intensive staining for A20 (Fig. 4j, n), while the strial basal cells, spiral ligament fibrocytes, and outer hair cells demonstrated mild staining for A20 (Fig. 4d, n). The strial basal cells, spiral ganglion cells, and inner pillar cells of Corti’s organ exhibited intensive staining for RNF11, while the spiral ligament fibrocytes, hair cells, and outer pillar cells displayed mild staining for RNF11 (Fig. 4h, l, p). The Deiters’ cells showed extremely weak staining for RNF11 (Fig. 4p). In the cochlear lateral wall, 0.4 % AgNPs enhanced the staining of A20 (*p* < 0.05 in the first and second turns and *p* > 0.05 in the third turn at the strial basal cells, *p* < 0.05 in the first and third turns and *p* < 0.01 in the second turn at the spiral ligament fibrocytes, one-way ANOVA) and RNF11 (*p* > 0.05 in the first and third turns and *p* < 0.05 in the second turn at the strial basal cells, *p* < 0.01 in the first turn and *p* < 0.05 in the second and third turns at the spiral ligament fibrocytes, one-way ANOVA) remarkably in the strial basal cells and spiral ligament fibrocytes that were independent of the cochlear turn (Fig. 4a–c, e–g) (*p* > 0.05, one-way ANOVA). In Corti’s organ, 0.4 % AgNPs increased A20 staining in the outer hair cells and Deiters’ cells (Fig. 4m) and RNF11 staining in the outer pillar cells and Deiters’ cells (Fig. 4o). In the spiral ganglion cells and capillary endothelial cells, 0.4 % AgNPs did not alter the staining of A20 and RNF11 in all turns (Fig. 4i, k) (*p* > 0.05, post hoc test). 0.02 % AgNPs had no influence on the staining of A20 and RNF11 in the aforementioned cells in all turns (images not shown) (*p* > 0.05, post hoc test).

There were no positive staining in the negative control slides (Additional file 12: Figure S12). The unchanged molecules in the rat cochlea exposed to AgNPs were summarized in Table 1.

**Table 1:** Unchanged molecules in the rat cochlea exposed to AgNPs

Functions/properties	Molecules
Cell recruitment	CD44
Innate immunity	TLR2
Chemotaxis	MCP2
Tight junction-associated proteins	VCAM1, Rac1, and MLC
Cellular signaling transduction	Erk1/2, JNK, and p38
Inflammation	IL-1β, TNF-α, TNFR1, TNFR2
Anti-inflammation	IL-10 and TGF-β

*MLC* myosin light chain

## Discussion

The current study showed that 0.4 % AgNPs but not 0.02 % AgNPs upregulated the expressions of CD68, TLR4, MCP1, A20, and RNF11 in the strial basal cells, spiral ligament fibrocytes, and non-sensory supporting cells of Corti’s organ. 0.4 % AgNPs had no effect on CD44, TLR2, MCP2, Rac1, myosin light chain, VCAM1, Erk1/2, JNK, p38, IL-1β, TNF-α, TNFR1, TNFR2, IL-10, or TGF-β. The toxicological mechanism of AgNPs is unclear. The Ag^+^ released from AgNPs was thought to be an important mediator involved in the pathological process associated with AgNPs exposure [35]. However, this is actually doubtful because no Ag^+^ remains in either animal or human body after reacting with the Cl^−^ and forming AgCl. The IC_50_ for AgNO_3_ was lower than that for AgNPs [1]. Our unpublished data demonstrated that AgCl did not cause any hearing loss at the second day through the seventh day post-intratympanic injection at the saturated concentration (520 μg/100 g). Therefore, our hypothesis is that the cytokine alteration in the current study is resulted from intact AgNPs rather than the disassociated Ag^+^.

Increasing evidence demonstrate that the inner ear is an active immune organ rather than an ‘immunologically privileged organ’ that was generally accepted previously [36]. Cochlear lateral wall including the stria vascularis and spiral ligament has been reported as the primary site harbouring macrophages in the inner ear of human and mouse [37, 38]. In the current study, cells that showed mild staining for CD68 without ramified morphology were identified in the stria vascularis and spiral ligament of rat cochlea exposed to dH_2_O, suggesting that the rat cochlea did not have typical tissue-resident macrophages and might have a different immune mechanism from the one in human. Macrophages were reportedly recruited into murine cochlea exposed to noise and ototoxic drugs [39–42]. The current study detected a sparse appearance of ramified CD68-positive cells in the spiral ligament and mononuclear cells in the modiolus of cochlea exposed to 0.4 % AgNPs, implying that either the rat cochlea possessed a different innate immune system from the mouse or the AgNPs triggered different signaling pathways from noise and conventional ototoxic drugs. The sole upregulation of MCP1 without sufficient cooperation with other molecules such as CD44, Rac1, myosin light chain, and VCAM1 might be the reason for failure in recruiting abundant macrophages into the cochlea [43–45]. Moreover, the unchanged levels of Erk1/2, JNK, and p38 did not provide the molecular basis for the adhesion and migration of monocytes [46]. Instead, the expressions of CD68 in the strial basal cells, spiral ligament fibrocytes, and non-sensory supporting cells of Corti’s organ were significantly upregulated after 0.4 % AgNPs exposure.

The upregulated CD68 might confer macrophage-like functions on the strial basal cells and spiral ligament fibrocytes and enhance the immune activities of non-sensory supporting cells of Corti’s organ. Non-sensory supporting cells of Corti’s organ are indicated as microglia-like cells and may determine the fate of the auditory sensory epithelium because microglia are believed to be macrophages in the central nervous system and play an irreplaceable role in immune surveillance [47–49]. CD68 was reportedly involved in vesicular trafficking to deliver the lipids to their proper intracellular compartments [50]. The current study suggested that CD68 might be implicated in the activation of TLR4 via caveolae trafficking operated by lipid raft and caveolin-1 phosphorylation [51]. Previous research indicated that AgNPs induced the accumulation of hyaluronan, the substrate of TLR4, in the cochlea [3]. TLR4 was also upregulated in the cochlea exposed to 0.4 % AgNPs in the current study. Theoretically, TLR4 activation triggers the NF-κB signaling pathway and finally upregulates the expressions of inflammatory cytokines including IL-1β, TNF-α, and its receptors TNFR1 and TNFR2. However, neither the downstream cytokines of macrophages nor TLR4 activation was upregulated in the cochlea exposed to AgNPs. Although it was unlikely that these pathways were never activated, it was predictable that certain cytokines were upregulated at an early stage but suppressed afterwards. This possibility was supported by previous studies showing that AgNPs caused reversible changes to the permeability of biological barriers in the rat inner ear and transient hearing loss that partially recovered as of the seventh day [1, 3].

A20, in the context of RNF11, has been shown to inhibit TLR-mediated inflammatory response, and it induced NF-κB signaling pathway [16, 17]. The current study showed that A20 and RNF11 were significantly upregulated in the strial basal cells, spiral ligament fibrocytes, and non-sensory supporting cells of Corti’s organ of the cochlea exposed to 0.4 % AgNPs, suggesting that A20 and RNF11 might play roles in maintaining cochlear homeostasis and thus preserving hearing [1, 3]. However, the incomplete hearing recovery in the high-frequency range in the AgNP-exposed ear suggested that the protective effects of A20 and RNF11 might be limited.

## Conclusions

AgNPs might confer macrophage-like functions on the strial basal cells and spiral ligament fibrocytes and enhance the immune activities of non-sensory supporting cells of Corti’s organ through the upregulation of CD68, which might be involved in TLR4 activation. A20 and RNF11 played roles in maintaining cochlear homeostasis via negative regulation of the expressions of inflammatory cytokines. The current study suggested that the rat cochlea might have a different immune mechanism from the one in human and mouse.

## Additional files

Additional file 1: Figure S1. CD44^+^ cells in the rat cochlea 7 days post-intratympanic injection of 0.4 % AgNPs shown by immunofluorescence confocal microscopy or immunohistochemistry. In the cochleae exposed to dH_2_O, the strial intermediate cells (SIMCs), strial basal cells (SBCs), spiral ligament fibrocytes (SLFs), spiral ganglion cells (SGCs), and outer hair cells (OHCs), pillar cells (PCs), and Deiters’ cells (DCs) of Corti’s organ (CO) showed intensive staining (B, D, and F), while the inner hair cells (IHCs) demonstrated mild staining (F). 0.4 % AgNPs had no influence on the staining in the SIMCs, SBCs, SLFs, SGCs, and CO (A, C, and E). Comparisons of staining intensity are shown in G and H. Scale bar = 50 μm in A–D and 20 μm in E and F. (JPG 4396 kb) Additional file 2: Figure S2. TLR2^+^ cells in the rat cochlea 7 days post-intratympanic injection of 0.4 % AgNPs shown by immunofluorescence confocal microscopy or immunohistochemistry. In the cochleae exposed to dH_2_O, the strial basal cells (SBCs), spiral ligament fibrocytes (SLFs) (mainly type II), spiral ganglion cells (SGCs), and inner hair cells (IHCs) and pillar cells (PCs) of Corti’s organ (CO) showed intensive staining (B, D, and F), while the outer hair cells (OHCs) and Deiters’ cells (DCs) demonstrated extremely weak staining (F). 0.4 % AgNPs enhanced the staining in the OHCs and DCs but not in the SBCs, SLFs, SGCs, IHCs, and PCs (A, C, and E). Comparisons of staining intensity are shown in G and H. Scale bar = 50 μm in A–D and 20 μm in E and F. (JPG 4393 kb) Additional file 3: Figure S3. MCP2^+^ cells in the rat cochlea 7 days post-intratympanic injection of 0.4 % AgNPs shown by immunofluorescence confocal microscopy or immunohistochemistry. In the cochleae exposed to dH_2_O, the strial basal cells (SBCs), spiral ligament fibrocytes (SLFs), spiral ganglion cells (SGCs), and hair cells (HCs), pillar cells (PCs), and Deiters’ cells (DCs) of Corti’s organ (CO) showed intensive staining (B, D, and F). 0.4 % AgNPs had no influence on the staining in the SBCs, SLFs, SGCs, and CO (A, C, and E). Comparisons of staining intensity are shown in G and H. Scale bar = 50 μm in A–D and 20 μm in E and F. (JPG 4614 kb) Additional file 4: Figure S4. Rac1^+^ cells in the rat cochlea 7 days post-intratympanic injection of 0.4 % AgNPs shown by immunofluorescence confocal microscopy or immunohistochemistry. In the cochleae exposed to dH_2_O, the strial intermediate cells (SIMCs), strial basal cells (SBCs), spiral ganglion cells (SGCs), and hair cells (HCs), pillar cells (PCs), and Deiters’ cells (DCs) of Corti’s organ (CO) showed intensive staining (B, D, and F), while the spiral ligament fibrocytes (SLFs) (mainly type II) demonstrated moderate staining (B). 0.4 % AgNPs had no influence on the staining in the SIMCs, SBCs, SLFs, SGCs, and CO (A, C, and E). Comparisons of staining intensity are shown in G and H. Scale bar = 50 μm in A–D and 20 μm in E and F. (JPG 4501 kb) Additional file 5: Figure S5. Myosin light chain positively stained cells in the rat cochlea 7 days post-intratympanic injection of 0.4 % AgNPs shown by immunofluorescence confocal microscopy or immunohistochemistry. In the cochleae exposed to dH_2_O, the spiral ganglion cells (SGCs) and inner pillar cells (IPCs) of Corti’s organ (CO) showed moderate staining (D and F), while the inner hair cells (IHCs), outer pillar cells (OPCs), outer hair cells (OHCs), and Deiters’ cells (DCs) demonstrated mild staining (F). The strial basal cells (SBCs) and spiral ligament fibrocytes (SLFs) exhibited extremely weak staining (B). 0.4 % AgNPs had no influence on the staining in the SBCs, SLFs, SGCs, and CO (A, C, and E). Scale bar = 50 μm in A–D and 20 μm in E and F. (JPG 4602 kb) Additional file 6: Figure S6. VCAM1^+^ cells in the rat cochlea 7 days post-intratympanic injection of 0.4 % AgNPs shown by immunofluorescence confocal microscopy or immunohistochemistry. In the cochleae exposed to dH_2_O, the strial basal cells (SBCs), spiral ligament fibrocytes (SLFs), spiral ganglion cells (SGCs), and hair cells (HCs), pillar cells (PCs), and Deiters’ cells (DCs) of Corti’s organ (CO) showed extremely weak staining (B, D, and F). 0.4 % AgNPs had no influence on the staining in the SBCs, SLFs, SGCs, and CO (A, C, and E). Scale bar = 30 μm. (JPG 4582 kb) Additional file 7: Figure S8. JNK^+^ and p38^+^ cells in the rat cochlea 7 days post-intratympanic injection of 0.4 % AgNPs shown by immunofluorescence confocal microscopy or immunohistochemistry. In the cochleae exposed to dH_2_O, the strial basal cells (SBCs), spiral ligament fibrocytes (SLFs), spiral ganglion cells (SGCs), and hair cells (HCs), pillar cells (PCs), and Deiters’ cells (DCs) of Corti’s organ (CO) showed extremely weak staining for JNK (B, F, and J) and p38 (D, H, and L). 0.4 % AgNPs had no influence on the staining of JNK (A, E, and I) and p38 (C, G, and K) in the SBCs, SLFs, SGCs, and CO. Scale bar = 50 μm in A–H and 20 μm in I–L. (JPG 4719 kb) Additional file 8: Figure S7. Erk1/2^+^ cells in the rat cochlea 7 days post-intratympanic injection of 0.4 % AgNPs shown by immunofluorescence confocal microscopy or immunohistochemistry. In the cochleae exposed to dH_2_O, the strial intermediate cells (SIMCs), strial basal cells (SBCs), spiral ligament fibrocytes (SLFs), spiral ganglion cells (SGCs), and hair cells (HCs), pillar cells (PCs), and Deiters’ cells (DCs) of Corti’s organ (CO) showed intensive staining (B, D, and F). 0.4 % AgNPs had no influence on the staining in the SIMCs, SBCs, SLFs, SGCs, and CO (A, C, and E). Comparisons of staining intensity are shown in G and H. Scale bar = 50 μm in A–D and 20 μm in E and F. (JPG 4418 kb) Additional file 9: Figure S9. TNF-α^+^, TNFR1^+^, and TNFR2^+^ cells in the rat cochlea 7 days post-intratympanic injection of 0.4 % AgNPs shown by immunofluorescence confocal microscopy or immunohistochemistry. In the cochleae exposed to dH_2_O, the spiral ganglion cells (SGCs), inner hair cells (IHCs), and inner pillar cells (IPCs) of Corti’s organ (CO) showed mild staining for TNF-α (H and N), while the strial basal cells (SBCs), spiral ligament fibrocytes (SLFs), outer pillar cells (OPCs), outer hair cells (OHCs), and Deiters’ cells (DCs) demonstrated extremely weak staining for TNF-α (B and N). The strial intermediate cells (SIMCs), SBCs, and SGCs exhibited mild staining for TNFR1 (D and J), while the SLFs, hair cells (HCs), pillar cells (PCs), and DCs displayed extremely weak staining for TNFR1 (D and P). The SIMCs and SBCs showed mild staining for TNFR2 (F), while the SLFs, SGCs, HCs, PCs, and DCs showed extremely weak staining for TNFR2 (F, L, and R). 0.4 % AgNPs had no influence on the staining of TNF-α (A, G, and M), TNFR1 (C, I, and O), and TNFR2 (E, K, and Q) in the SIMCs, SBCs, SLFs, SGCs, and CO. Scale bar = 30 μm. (JPG 4651 kb) Additional file 10: Figure S10. IL-1β^+^ cells in the rat cochlea 7 days post-intratympanic injection of 0.4 % AgNPs shown by immunofluorescence confocal microscopy or immunohistochemistry. In the cochleae exposed to dH_2_O, the strial basal cells (SBCs), spiral ganglion cells (SGCs), and pillar cells (PCs) of Corti’s organ (CO) showed intensive staining, while the spiral ligament fibrocytes (SLFs) (mainly type II) and inner hair cells (IHCs) demonstrated mild staining (B, D, and F). The outer hair cells (OHCs) and Deiters’ cells (DCs) exhibited extremely weak staining (F). 0.4 % AgNPs had no influence on the staining in the SBCs, SLFs, SGCs, and CO (A, C, and E). Comparisons of staining intensity are shown in G and H. Scale bar = 50 μm in A–D and 20 μm in E and F. (JPG 4326 kb) Additional file 11: Figure S11. IL-10^+^ and TGF-β^+^ cells in the rat cochlea 7 days post-intratympanic injection of 0.4 % AgNPs shown by immunofluorescence confocal microscopy or immunohistochemistry. In the cochleae exposed to dH_2_O, the spiral ganglion cells (SGCs) showed intensive staining for IL-10 (F), while the pillar cells (PCs) of Corti’s organ (CO) demonstrated mild staining for IL-10 (J). The strial basal cells (SBCs), spiral ligament fibrocytes (SLFs), hair cells (HCs), and Deiters’ cells (DCs) exhibited extremely weak staining for IL-10 (B and J). The SGCs and PCs of CO displayed intensive staining for TGF-β (H and L), while the SBCs, SLFs, and inner hair cells (IHCs) demonstrated mild staining for TGF-β (D and L). The outer hair cells (OHCs) and DCs showed extremely weak staining for TGF-β (L). 0.4 % AgNPs had no influence on the staining of IL-10 (A, E, and I) and TGF-β (C, G, and K) in the SBCs, SLFs, SGCs, and CO. Comparisons of staining intensity are shown in M and N. Scale bar = 50 μm in A–H and 20 μm in I–L. (JPG 4356 kb) Additional file 12: Figure S12. Negative control. Scale bar = 50 μm in A and C, 20 μm in E, and 30 μm in B, D, and F. (JPG 3915 kb)

## Abbreviations

AgNPs: silver nanoparticles
DAMP: danger/damage-associated molecular pattern
Erk1/2: extracellular signal-regulated kinases 1/2
IL-10: interleukin-10
IL-1β: interleukin-1β
JNK1/2/3: c-Jun N-terminal kinases 1/2/3
MCPs: monocyte chemoattractant proteins
NF-κB: nuclear factor-κB
PAMP: pathogen-associated molecular pattern
PRR: pattern recognition receptor
RNF11: RING finger protein 11
SBCs: strial basal cells
SGCs: spiral ganglion cells
SLFs: spiral ligament fibrocytes
TGF-β: transforming growth factor-β
TLR2/4: toll-like receptors 2/4
TNFRs: tumour necrosis factor receptors
TNF-α: tumour necrosis factor-α
VCAM1: vascular cell adhesion molecule 1
