# Supplementary figures and images for: Involvement of Ubiquitin-Editing Protein A20 in Modulating Inflammation in Rat Cochlea Associated with Silver Nanoparticle-Induced CD68 Upregulation and TLR4 Activation
Source: Nanoscale Res Lett. 2016 May 4;11:240. doi: 10.1186/s11671-016-1430-9 (PMC4854861; doi:10.1186/s11671-016-1430-9)

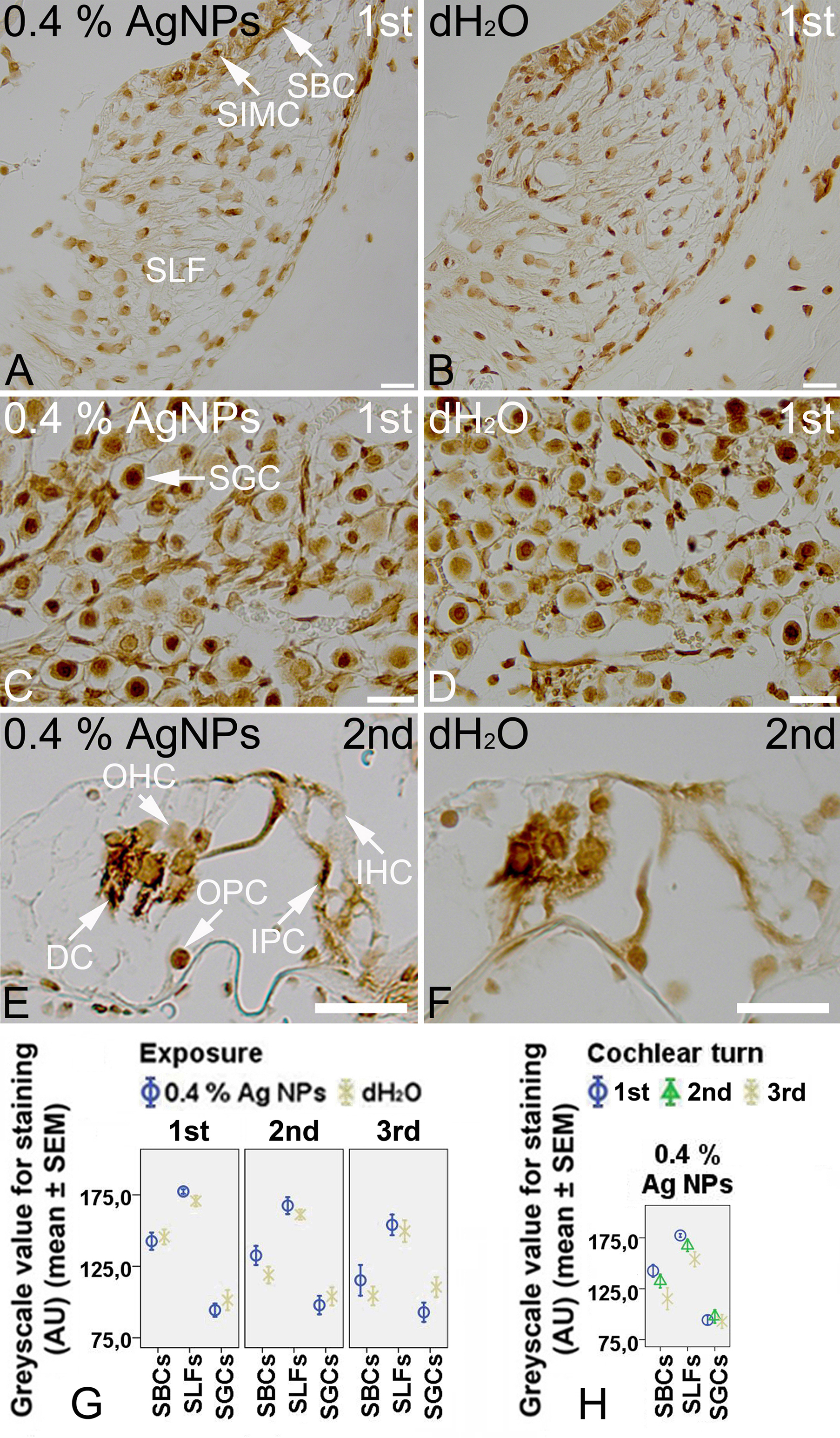

Supplement: Additional file 1: Figure S1. — CD44+ cells in the rat cochlea 7 days post-intratympanic injection of 0.4 % AgNPs shown by immunofluorescence confocal microscopy or immunohistochemistry. In the cochleae exposed to dH2O, the strial intermediate cells (SIMCs), strial basal cells (SBCs), spiral ligament fibrocytes (SLFs), spiral ganglion cells (SGCs), and outer hair cells (OHCs), pillar cells (PCs), and Deiters’ cells (DCs) of Corti’s organ (CO) showed intensive staining (B, D, and F), while the inner hair cells (IHCs) demonstrated mild staining (F). 0.4 % AgNPs had no influence on the staining in the SIMCs, SBCs, SLFs, SGCs, and CO (A, C, and E). Comparisons of staining intensity are shown in G and H. Scale bar = 50 μm in A–D and 20 μm in E and F. (JPG 4396 kb) [file 11671_2016_1430_MOESM1_ESM.jpg]

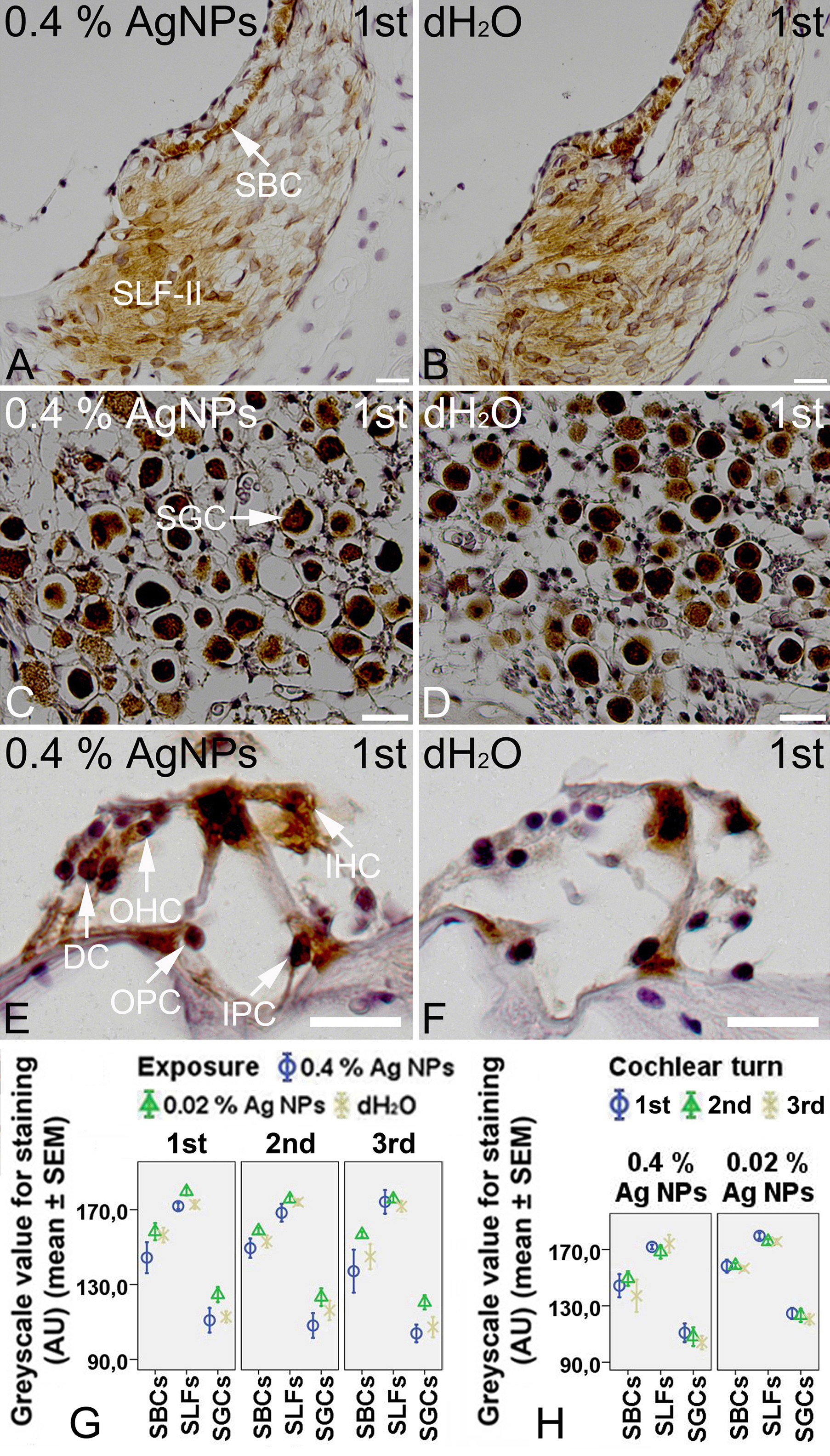

Supplement: Additional file 2: Figure S2. — TLR2+ cells in the rat cochlea 7 days post-intratympanic injection of 0.4 % AgNPs shown by immunofluorescence confocal microscopy or immunohistochemistry. In the cochleae exposed to dH2O, the strial basal cells (SBCs), spiral ligament fibrocytes (SLFs) (mainly type II), spiral ganglion cells (SGCs), and inner hair cells (IHCs) and pillar cells (PCs) of Corti’s organ (CO) showed intensive staining (B, D, and F), while the outer hair cells (OHCs) and Deiters’ cells (DCs) demonstrated extremely weak staining (F). 0.4 % AgNPs enhanced the staining in the OHCs and DCs but not in the SBCs, SLFs, SGCs, IHCs, and PCs (A, C, and E). Comparisons of staining intensity are shown in G and H. Scale bar = 50 μm in A–D and 20 μm in E and F. (JPG 4393 kb) [file 11671_2016_1430_MOESM2_ESM.jpg]

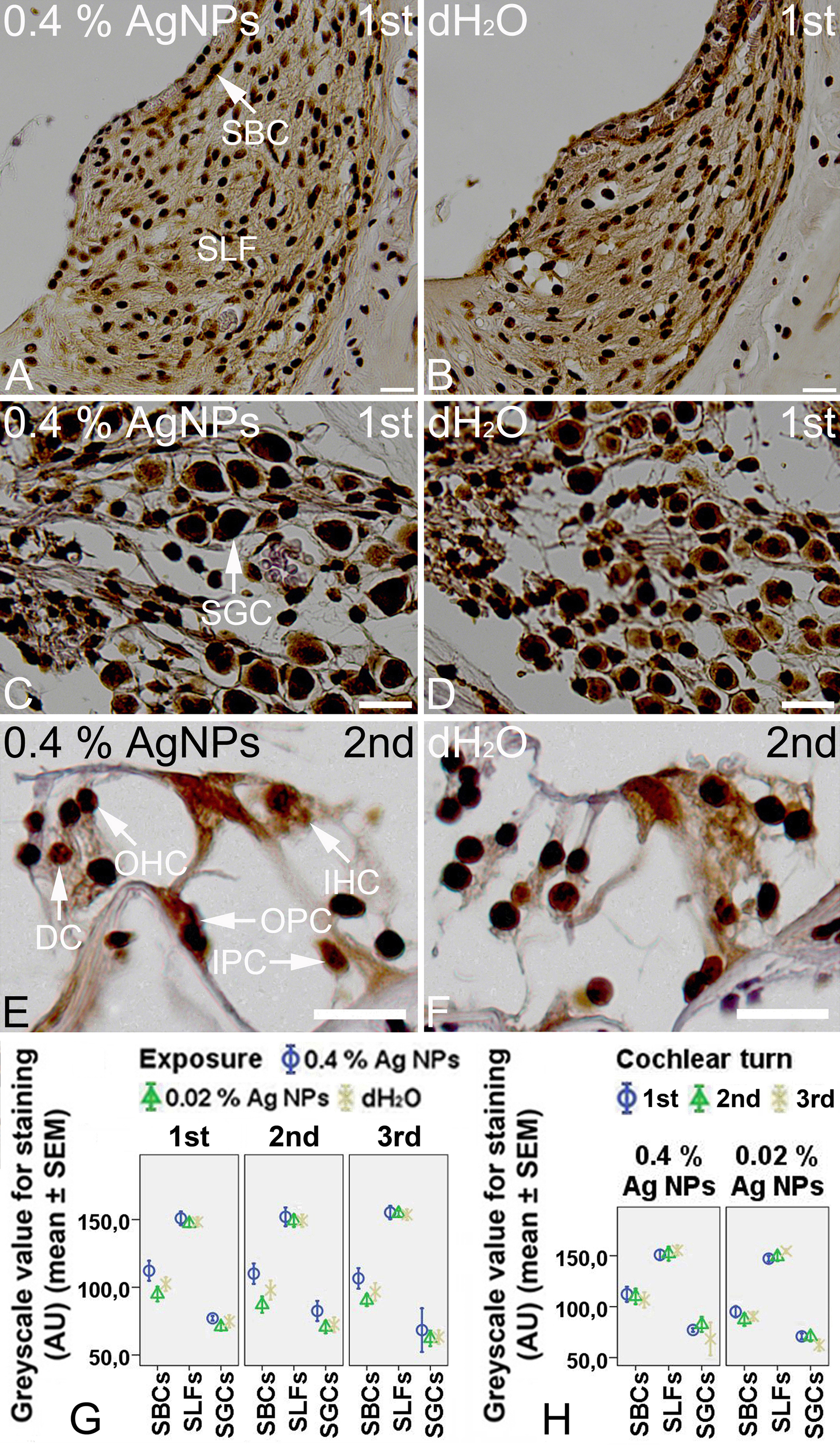

Supplement: Additional file 3: Figure S3. — MCP2+ cells in the rat cochlea 7 days post-intratympanic injection of 0.4 % AgNPs shown by immunofluorescence confocal microscopy or immunohistochemistry. In the cochleae exposed to dH2O, the strial basal cells (SBCs), spiral ligament fibrocytes (SLFs), spiral ganglion cells (SGCs), and hair cells (HCs), pillar cells (PCs), and Deiters’ cells (DCs) of Corti’s organ (CO) showed intensive staining (B, D, and F). 0.4 % AgNPs had no influence on the staining in the SBCs, SLFs, SGCs, and CO (A, C, and E). Comparisons of staining intensity are shown in G and H. Scale bar = 50 μm in A–D and 20 μm in E and F. (JPG 4614 kb) [file 11671_2016_1430_MOESM3_ESM.jpg]

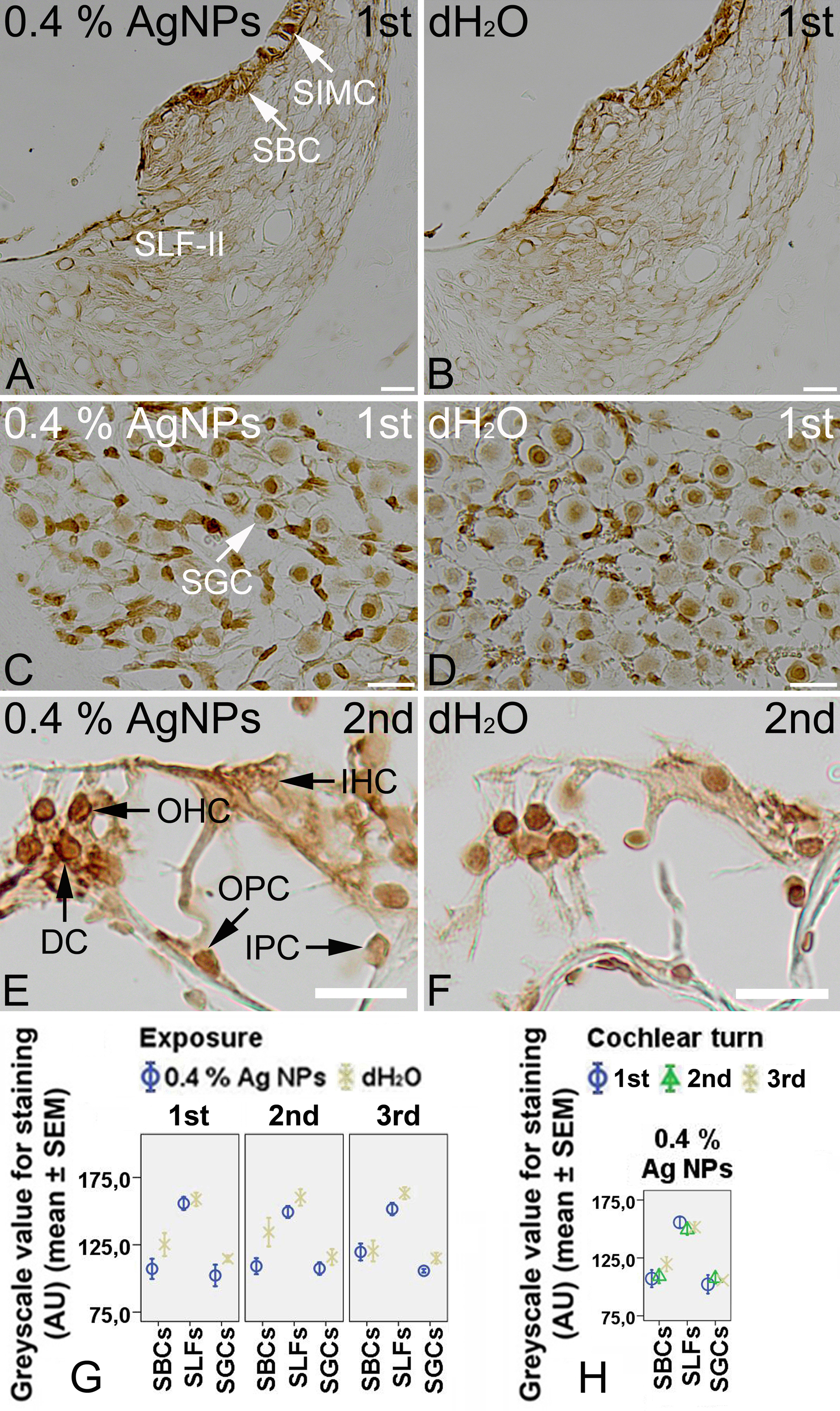

Supplement: Additional file 4: Figure S4. — Rac1+ cells in the rat cochlea 7 days post-intratympanic injection of 0.4 % AgNPs shown by immunofluorescence confocal microscopy or immunohistochemistry. In the cochleae exposed to dH2O, the strial intermediate cells (SIMCs), strial basal cells (SBCs), spiral ganglion cells (SGCs), and hair cells (HCs), pillar cells (PCs), and Deiters’ cells (DCs) of Corti’s organ (CO) showed intensive staining (B, D, and F), while the spiral ligament fibrocytes (SLFs) (mainly type II) demonstrated moderate staining (B). 0.4 % AgNPs had no influence on the staining in the SIMCs, SBCs, SLFs, SGCs, and CO (A, C, and E). Comparisons of staining intensity are shown in G and H. Scale bar = 50 μm in A–D and 20 μm in E and F. (JPG 4501 kb) [file 11671_2016_1430_MOESM4_ESM.jpg]

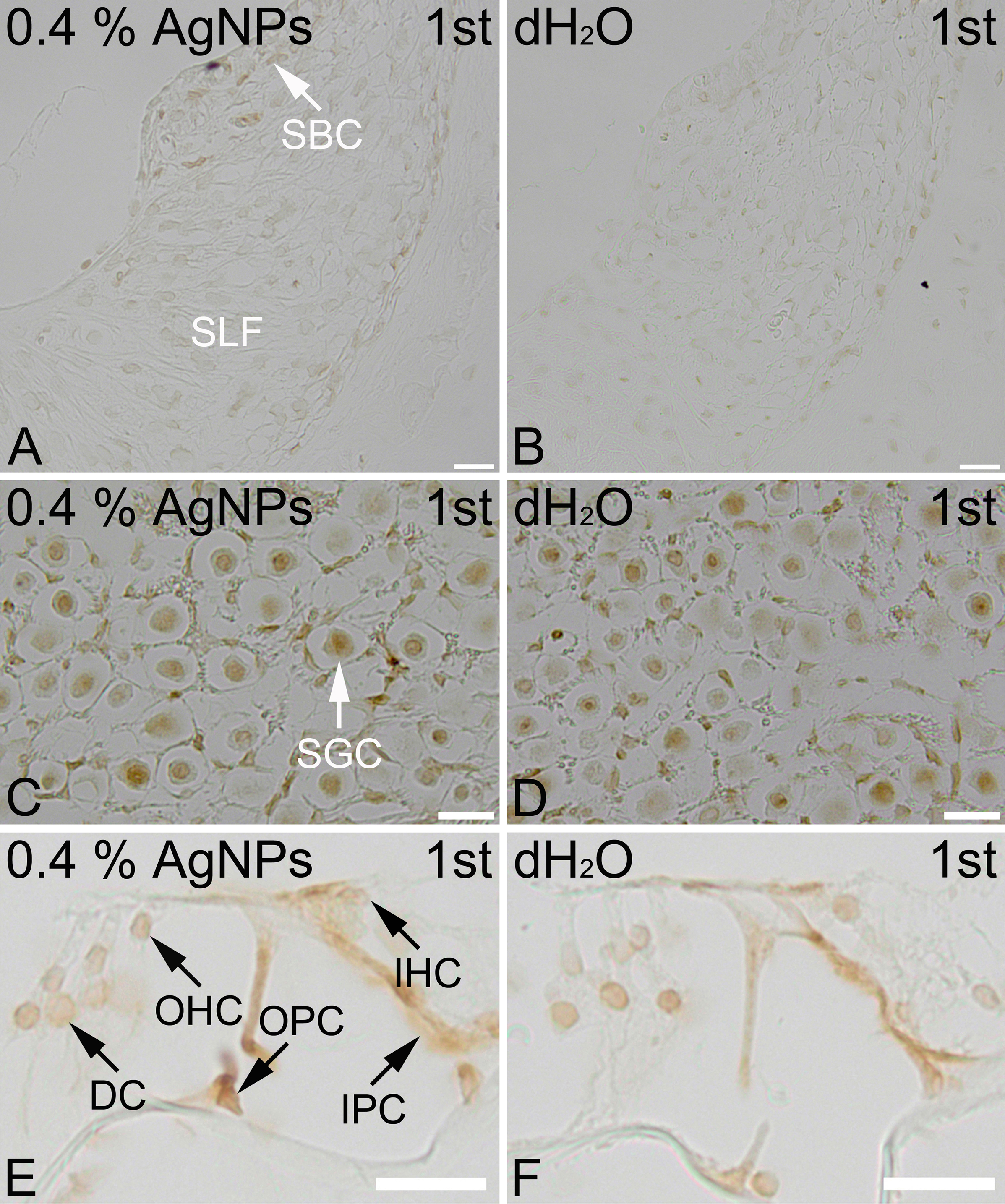

Supplement: Additional file 5: Figure S5. — Myosin light chain positively stained cells in the rat cochlea 7 days post-intratympanic injection of 0.4 % AgNPs shown by immunofluorescence confocal microscopy or immunohistochemistry. In the cochleae exposed to dH2O, the spiral ganglion cells (SGCs) and inner pillar cells (IPCs) of Corti’s organ (CO) showed moderate staining (D and F), while the inner hair cells (IHCs), outer pillar cells (OPCs), outer hair cells (OHCs), and Deiters’ cells (DCs) demonstrated mild staining (F). The strial basal cells (SBCs) and spiral ligament fibrocytes (SLFs) exhibited extremely weak staining (B). 0.4 % AgNPs had no influence on the staining in the SBCs, SLFs, SGCs, and CO (A, C, and E). Scale bar = 50 μm in A–D and 20 μm in E and F. (JPG 4602 kb) [file 11671_2016_1430_MOESM5_ESM.jpg]

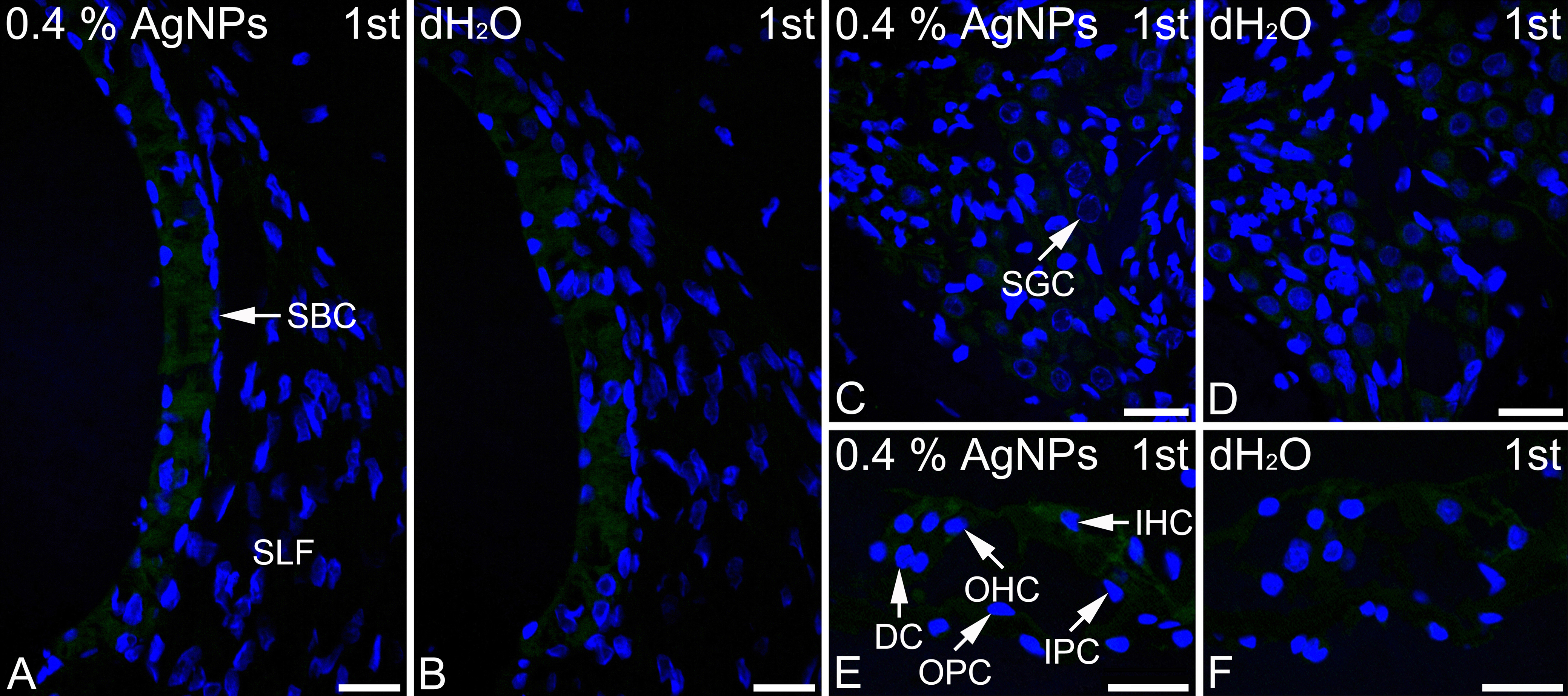

Supplement: Additional file 6: Figure S6. — VCAM1+ cells in the rat cochlea 7 days post-intratympanic injection of 0.4 % AgNPs shown by immunofluorescence confocal microscopy or immunohistochemistry. In the cochleae exposed to dH2O, the strial basal cells (SBCs), spiral ligament fibrocytes (SLFs), spiral ganglion cells (SGCs), and hair cells (HCs), pillar cells (PCs), and Deiters’ cells (DCs) of Corti’s organ (CO) showed extremely weak staining (B, D, and F). 0.4 % AgNPs had no influence on the staining in the SBCs, SLFs, SGCs, and CO (A, C, and E). Scale bar = 30 μm. (JPG 4582 kb) [file 11671_2016_1430_MOESM6_ESM.jpg]

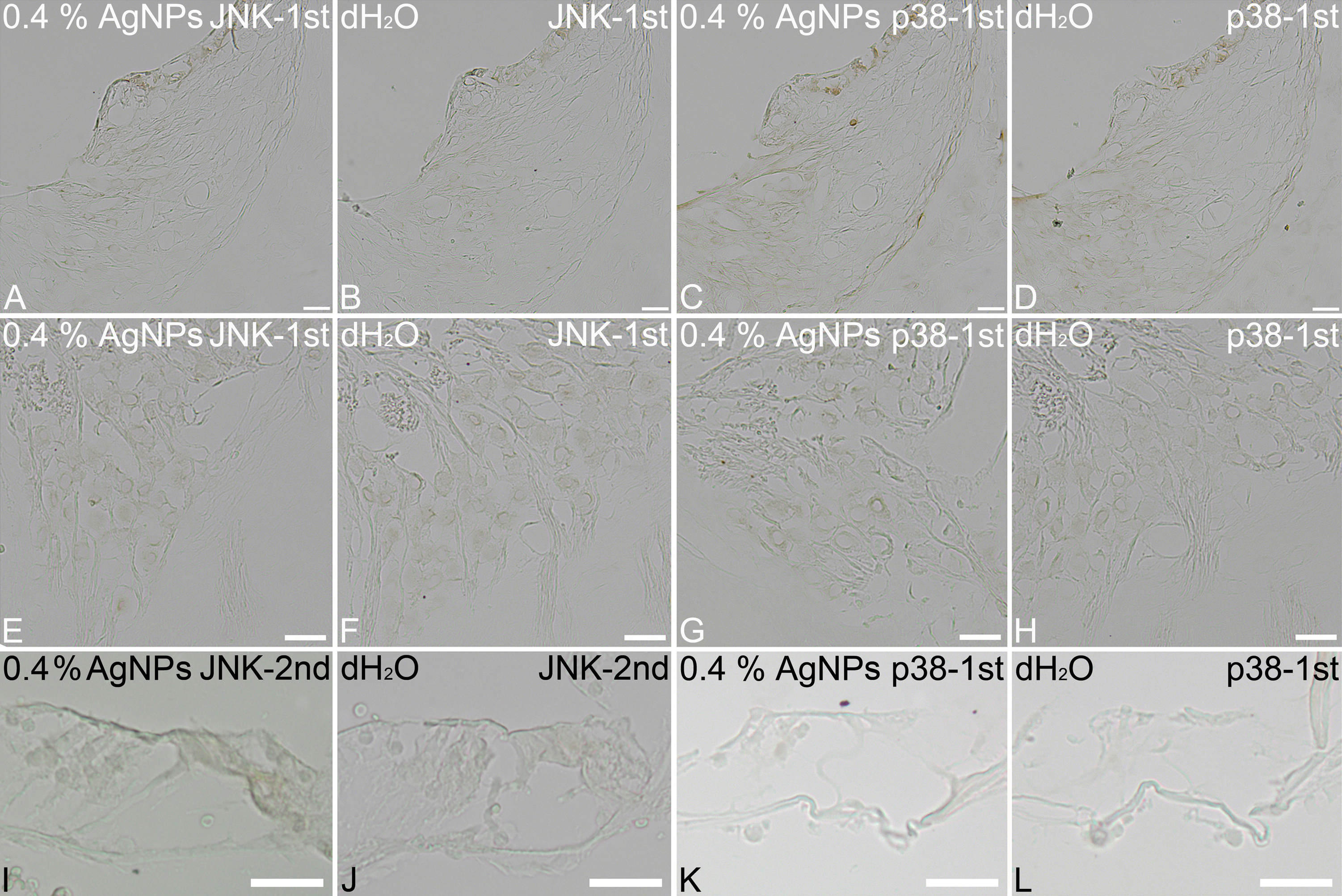

Supplement: Additional file 7: Figure S8. — JNK+ and p38+ cells in the rat cochlea 7 days post-intratympanic injection of 0.4 % AgNPs shown by immunofluorescence confocal microscopy or immunohistochemistry. In the cochleae exposed to dH2O, the strial basal cells (SBCs), spiral ligament fibrocytes (SLFs), spiral ganglion cells (SGCs), and hair cells (HCs), pillar cells (PCs), and Deiters’ cells (DCs) of Corti’s organ (CO) showed extremely weak staining for JNK (B, F, and J) and p38 (D, H, and L). 0.4 % AgNPs had no influence on the staining of JNK (A, E, and I) and p38 (C, G, and K) in the SBCs, SLFs, SGCs, and CO. Scale bar = 50 μm in A–H and 20 μm in I–L. (JPG 4719 kb) [file 11671_2016_1430_MOESM7_ESM.jpg]

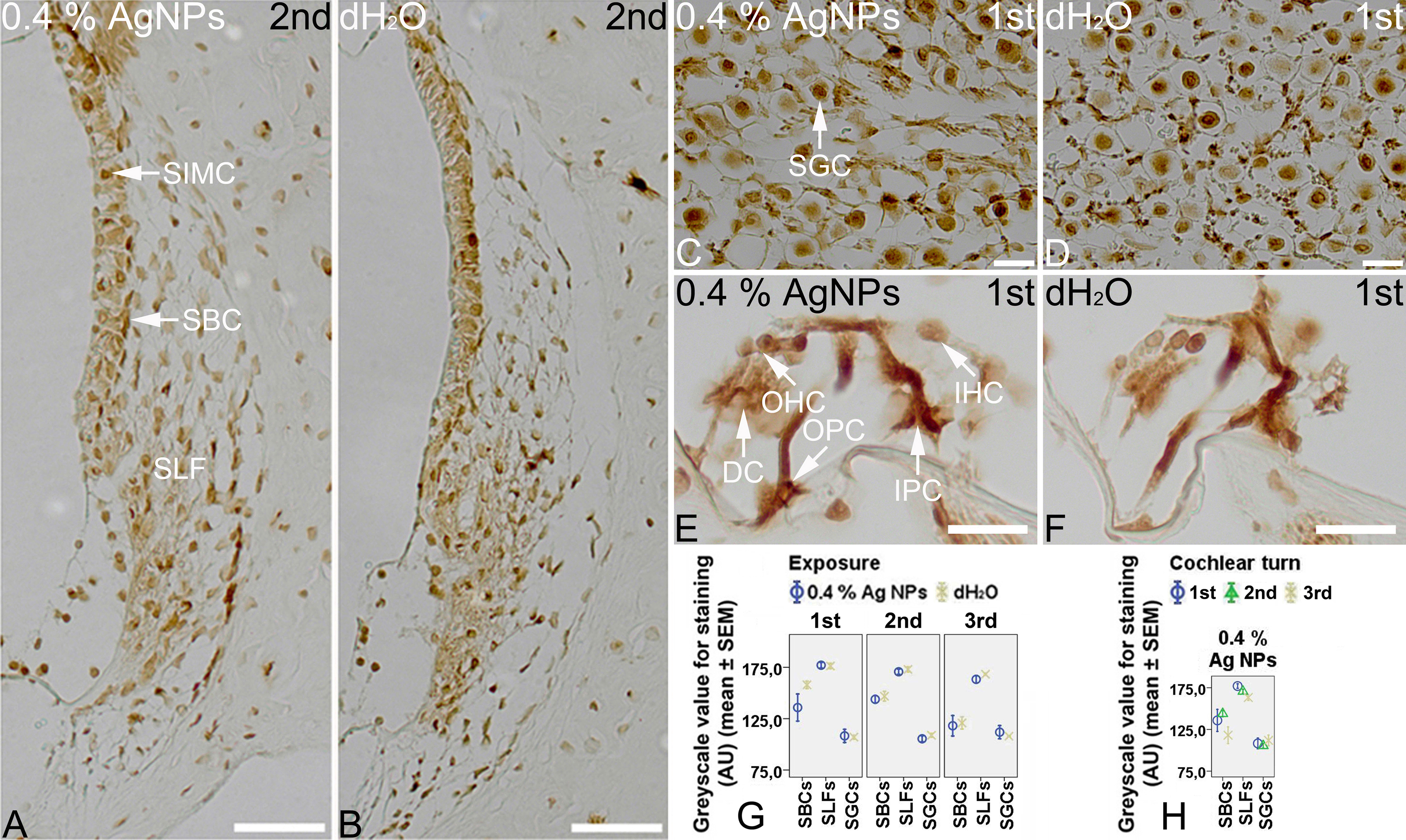

Supplement: Additional file 8: Figure S7. — Erk1/2+ cells in the rat cochlea 7 days post-intratympanic injection of 0.4 % AgNPs shown by immunofluorescence confocal microscopy or immunohistochemistry. In the cochleae exposed to dH2O, the strial intermediate cells (SIMCs), strial basal cells (SBCs), spiral ligament fibrocytes (SLFs), spiral ganglion cells (SGCs), and hair cells (HCs), pillar cells (PCs), and Deiters’ cells (DCs) of Corti’s organ (CO) showed intensive staining (B, D, and F). 0.4 % AgNPs had no influence on the staining in the SIMCs, SBCs, SLFs, SGCs, and CO (A, C, and E). Comparisons of staining intensity are shown in G and H. Scale bar = 50 μm in A–D and 20 μm in E and F. (JPG 4418 kb) [file 11671_2016_1430_MOESM8_ESM.jpg]

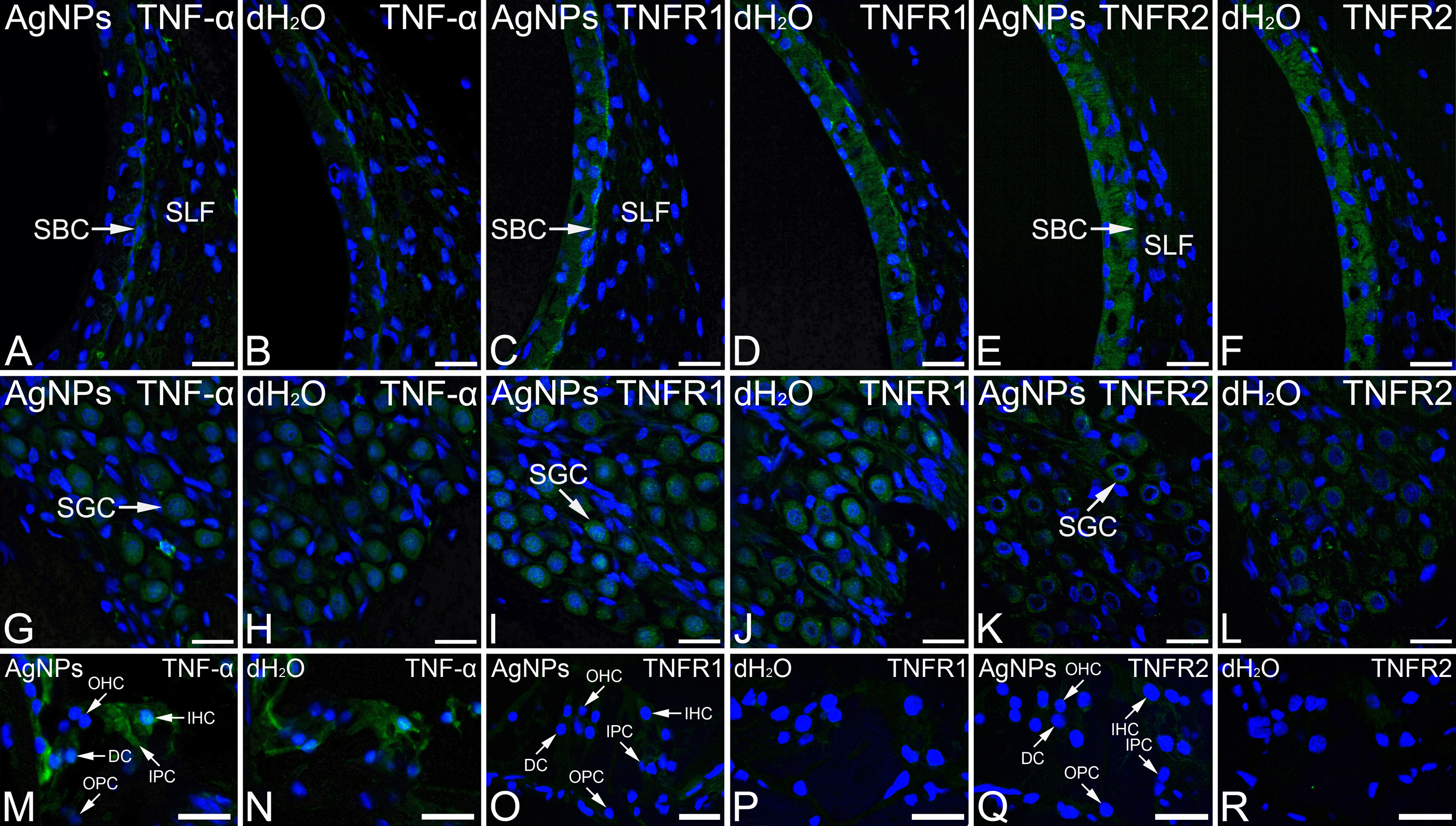

Supplement: Additional file 9: Figure S9. — TNF-α+, TNFR1+, and TNFR2+ cells in the rat cochlea 7 days post-intratympanic injection of 0.4 % AgNPs shown by immunofluorescence confocal microscopy or immunohistochemistry. In the cochleae exposed to dH2O, the spiral ganglion cells (SGCs), inner hair cells (IHCs), and inner pillar cells (IPCs) of Corti’s organ (CO) showed mild staining for TNF-α (H and N), while the strial basal cells (SBCs), spiral ligament fibrocytes (SLFs), outer pillar cells (OPCs), outer hair cells (OHCs), and Deiters’ cells (DCs) demonstrated extremely weak staining for TNF-α (B and N). The strial intermediate cells (SIMCs), SBCs, and SGCs exhibited mild staining for TNFR1 (D and J), while the SLFs, hair cells (HCs), pillar cells (PCs), and DCs displayed extremely weak staining for TNFR1 (D and P). The SIMCs and SBCs showed mild staining for TNFR2 (F), while the SLFs, SGCs, HCs, PCs, and DCs showed extremely weak staining for TNFR2 (F, L, and R). 0.4 % AgNPs had no influence on the staining of TNF-α (A, G, and M), TNFR1 (C, I, and O), and TNFR2 (E, K, and Q) in the SIMCs, SBCs, SLFs, SGCs, and CO. Scale bar = 30 μm. (JPG 4651 kb) [file 11671_2016_1430_MOESM9_ESM.jpg]

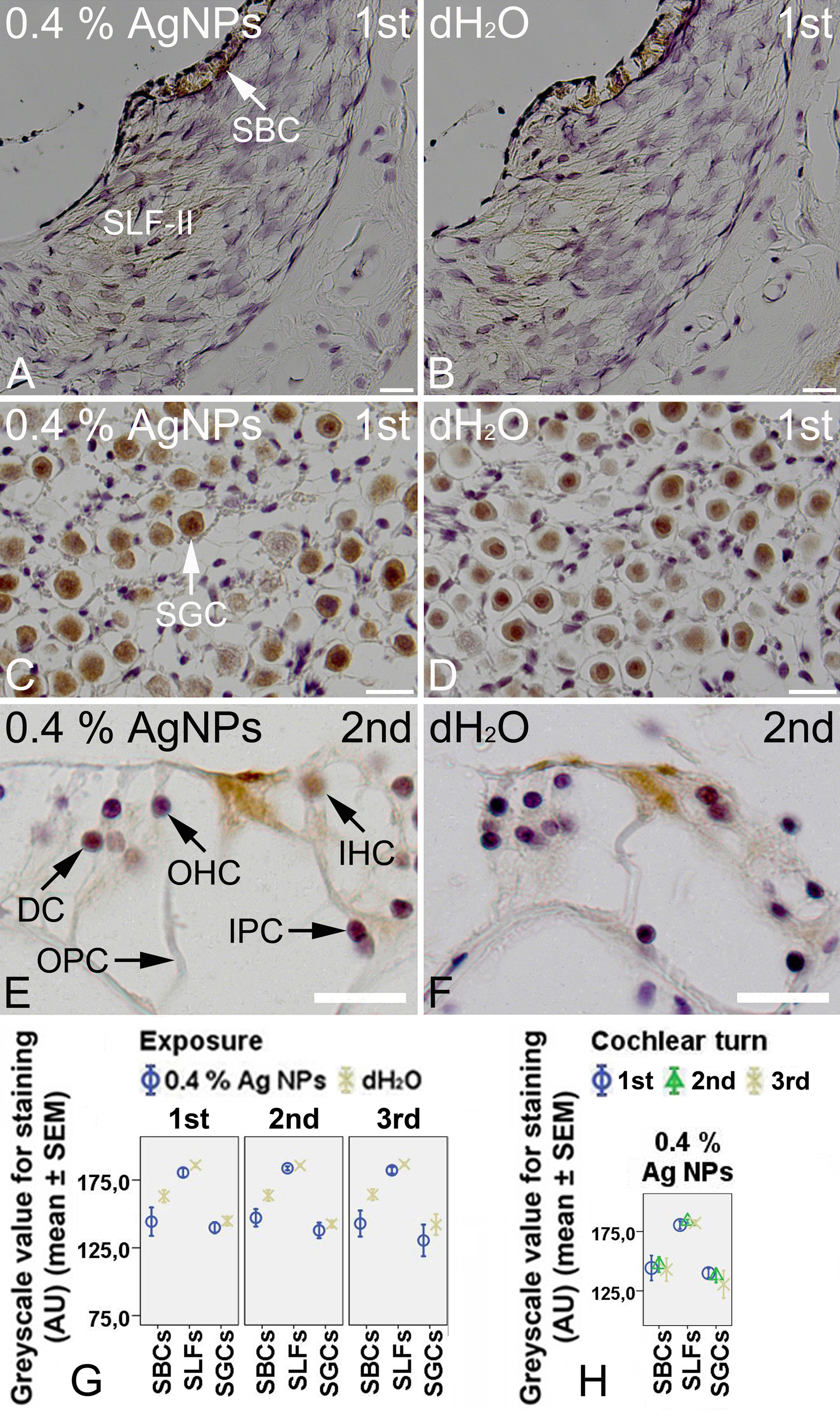

Supplement: Additional file 10: Figure S10. — IL-1β+ cells in the rat cochlea 7 days post-intratympanic injection of 0.4 % AgNPs shown by immunofluorescence confocal microscopy or immunohistochemistry. In the cochleae exposed to dH2O, the strial basal cells (SBCs), spiral ganglion cells (SGCs), and pillar cells (PCs) of Corti’s organ (CO) showed intensive staining, while the spiral ligament fibrocytes (SLFs) (mainly type II) and inner hair cells (IHCs) demonstrated mild staining (B, D, and F). The outer hair cells (OHCs) and Deiters’ cells (DCs) exhibited extremely weak staining (F). 0.4 % AgNPs had no influence on the staining in the SBCs, SLFs, SGCs, and CO (A, C, and E). Comparisons of staining intensity are shown in G and H. Scale bar = 50 μm in A–D and 20 μm in E and F. (JPG 4326 kb) [file 11671_2016_1430_MOESM10_ESM.jpg]

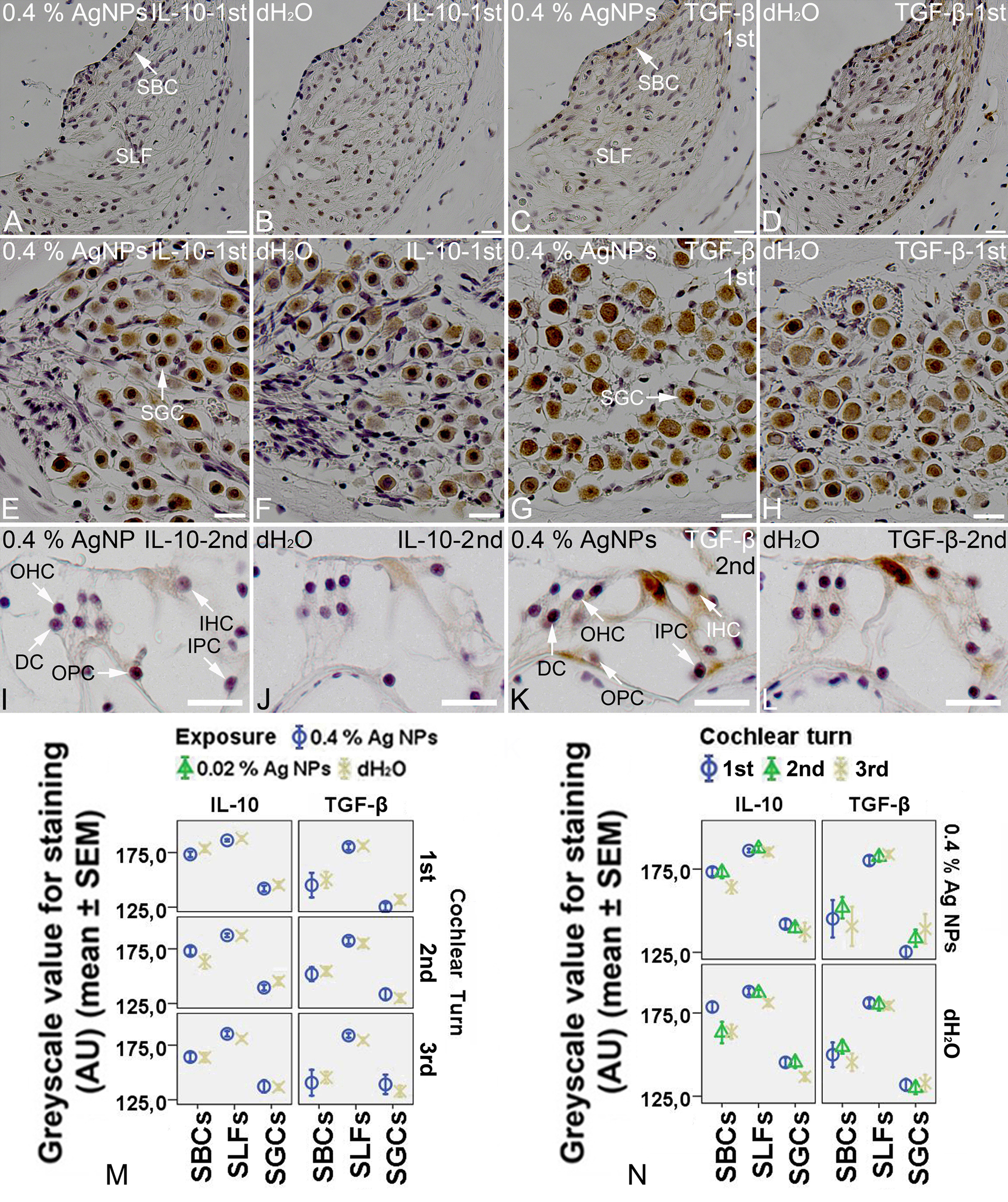

Supplement: Additional file 11: Figure S11. — IL-10+ and TGF-β+ cells in the rat cochlea 7 days post-intratympanic injection of 0.4 % AgNPs shown by immunofluorescence confocal microscopy or immunohistochemistry. In the cochleae exposed to dH2O, the spiral ganglion cells (SGCs) showed intensive staining for IL-10 (F), while the pillar cells (PCs) of Corti’s organ (CO) demonstrated mild staining for IL-10 (J). The strial basal cells (SBCs), spiral ligament fibrocytes (SLFs), hair cells (HCs), and Deiters’ cells (DCs) exhibited extremely weak staining for IL-10 (B and J). The SGCs and PCs of CO displayed intensive staining for TGF-β (H and L), while the SBCs, SLFs, and inner hair cells (IHCs) demonstrated mild staining for TGF-β (D and L). The outer hair cells (OHCs) and DCs showed extremely weak staining for TGF-β (L). 0.4 % AgNPs had no influence on the staining of IL-10 (A, E, and I) and TGF-β (C, G, and K) in the SBCs, SLFs, SGCs, and CO. Comparisons of staining intensity are shown in M and N. Scale bar = 50 μm in A–H and 20 μm in I–L. (JPG 4356 kb) [file 11671_2016_1430_MOESM11_ESM.jpg]

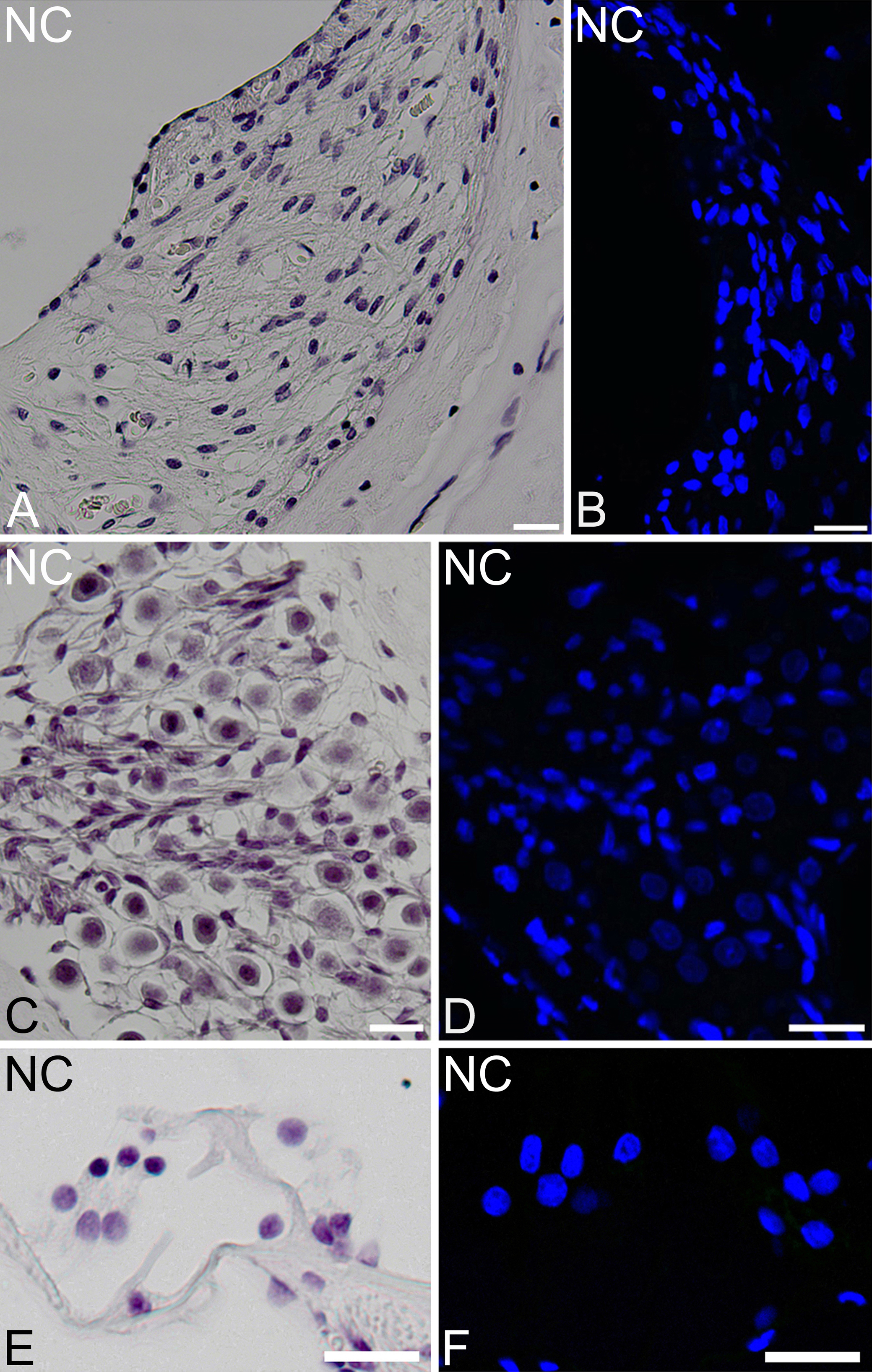

Supplement: Additional file 12: Figure S12. — Negative control. Scale bar = 50 μm in A and C, 20 μm in E, and 30 μm in B, D, and F. (JPG 3915 kb) [file 11671_2016_1430_MOESM12_ESM.jpg]
